# Two plant-associated *Bacillus velezensis* strains selected after genome analysis, metabolite profiling, and with proved biocontrol potential, were enhancing harvest yield of coffee and black pepper in large field trials

**DOI:** 10.3389/fpls.2023.1194887

**Published:** 2023-06-23

**Authors:** Le Thi Thanh Tam, Jennifer Jähne, Pham Thi Luong, Le Thi Phuong Thao, Le Mai Nhat, Christian Blumenscheit, Andy Schneider, Jochen Blom, Le Thi Kim Chung, Pham Le Anh Minh, Ha Minh Thanh, Trinh Xuan Hoat, Pham Cong Hoat, Tran Cao Son, Markus Weinmann, Stefanie Herfort, Joachim Vater, Nguyen Van Liem, Thomas Schweder, Peter Lasch, Rainer Borriss

**Affiliations:** ^1^Division of Pathology and Phyto-Immunology, Plant Protection Research Institute (PPRI), Ha Noi, Vietnam; ^2^Proteomics and Spectroscopy Unit (ZBS6), Center for Biological Threats and Special Pathogens, Robert Koch Institute, Berlin, Germany; ^3^Science and International Co-operation Department, Plant Protection Research Institute (PPRI), Ha Noi, Vietnam; ^4^Bioinformatics and Systems Biology, Justus-Liebig-Universität Giessen, Giessen, Germany; ^5^Institute for Preventive Medicine and Public Health, Hanoi Medical University, Ha Noi, Vietnam; ^6^Department of Biotechnology, Vietnam National University of Agriculture, Ha Noi, Vietnam; ^7^Department of Science and Technology for Economic Technical Branches, Ministry of Science and Technology (MOST), Hanoi, Vietnam; ^8^Laboratory of Food Toxicology and Allergens, National Institute for Food Control (NIFC), Ha Noi, Vietnam; ^9^Ernährungsphysiologie Der Kulturpflanzen, University of Hohenheim, Stuttgart, Germany; ^10^Institute of Marine Biotechnology e.V. (IMaB), Greifswald, Germany; ^11^Pharmaceutical Biotechnology, University of Greifswald, Greifswald, Germany; ^12^Institute of Biology, Humboldt University, Berlin, Germany

**Keywords:** *Bacillus velezensis*, phylogenomics, biocontrol plant pathogens, microbial biostimulant, Vietnamese agriculture, black pepper, coffee trees, crop yield

## Abstract

Elimination of chemically synthesized pesticides, such as fungicides and nematicides, in agricultural products is a key to successful practice of the Vietnamese agriculture. We describe here the route for developing successful biostimulants based on members of the *Bacillus subtilis* species complex. A number of endospore-forming Gram-positive bacterial strains with antagonistic action against plant pathogens were isolated from Vietnamese crop plants. Based on their draft genome sequence, thirty of them were assigned to the *Bacillus subtilis* species complex. Most of them were assigned to the species *Bacillus velezensis*. Whole genome sequencing of strains BT2.4 and BP1.2A corroborated their close relatedness to *B. velezensis* FZB42, the model strain for Gram-positive plant growth-promoting bacteria. Genome mining revealed that at least 15 natural product biosynthesis gene clusters (BGCs) are well conserved in all *B. velezensis* strains. In total, 36 different BGCs were identified in the genomes of the strains representing *B. velezensis, B. subtilis, Bacillus tequilensis*, and *Bacillus. altitudinis*. *In vitro* and *in vivo* assays demonstrated the potential of the *B. velezensis* strains to enhance plant growth and to suppress phytopathogenic fungi and nematodes. Due to their promising potential to stimulate plant growth and to support plant health, the *B. velezensis* strains TL7 and S1 were selected as starting material for the development of novel biostimulants, and biocontrol agents efficient in protecting the important Vietnamese crop plants black pepper and coffee against phytopathogens. The results of the large-scale field trials performed in the Central Highlands in Vietnam corroborated that TL7 and S1 are efficient in stimulating plant growth and protecting plant health in large-scale applications. It was shown that treatment with both bioformulations resulted in prevention of the pathogenic pressure exerted by nematodes, fungi, and oomycetes, and increased harvest yield in coffee, and pepper.

## 1 Introduction

Fruits from tropical tree crops, such as bananas, mangos, spices, coffee, and cacao, are widely traded and worldwide sought (Drenth and Guest, 2016). Coffee and black pepper are important crops in tropical, especially in Vietnamese agriculture. Coffee production was introduced to Vietnam by France around the year 1915. The cultivation of coffee is now a corner stone of the local economy. In 2022, the coffee area in Vietnam, which is mainly located in the Central Highland (Dak Lak province), amounted to 695.6 thousand hectares with the production of green coffee reaching 1,763.5 thousand tons per year (General Statistics Office – GSO, https://www.gso.gov.vn/en/). Robusta coffee (*Coffea canephora*) occupies most of the area (93%). The remaining part is used for cultivating the high-value Arabica coffee (*Coffea arabica*). The natural conditions in the Vietnamese mountain region, such as acidic soil, rain pattern and high elevation, provide a unique environment for Arabica coffee trees. Vietnamese coffee is exported to over 80 countries and territories. In 2018, Vietnam exported 1.9 million tons with an export turnover of 3.5 billion USD. This accounts for 14% of the market share and more than 10% of the global export value of green coffee, ranking second after Brazil (Rolshausen and Dzung, 2018).

Despite strong international competition, Vietnam made amazing progress in production of black pepper (*Piper nigrum*) fruits known as peppercorns, from nearly no-production in 1983 to the world number one today. In 2020, the area of ​​pepper reached 131.8 thousand hectares, of which 112.9 thousand hectares were harvested with an output of 270.2 thousand tons. Pepper is grown mainly in the Southeast region and the Central Highlands. Vietnam’s pepper output accounts for nearly 40% of this common daily spice worldwide (Vietnam Pepper Association (VPA), 2020).

However, in recent years harvest losses caused by pathogenic microorganisms, and root-knot forming nematodes have been steadily increasing (Tam et al., 2020; Jähne et al., 2023). A serious problem in Vietnamese agriculture is the occurrence of an aggressive wilt disease in coffee trees with symptoms as yellowing leaves, wilting foliage, and root rot (internally named ´YLRR´). The causative agent of the disease was identified as being the root-knot forming nematode *Meloidogyne* sp., often associated with the fungus *Fusarium oxysporum* (Nguyen et al., 2016). In addition, also the black pepper cultivation has been seriously affected by a number of diseases (Santos et al., 2023). Among them, the “quick wilt” or “quick death” disease caused by the root-knot nematode *Meloidogyne* spp, and the oomycete *Phytophthora palmivora* has a serious negative effect on black pepper growth and productivity (Nguyen et al., 2021). Widespread application of disease management strategies, based on chemically synthesized fungicides and nematicides, such as carbamate and organophosphorous, were found worldwide associated with increasing resistance of the target pathogens (Stukenbrock and Gurr, 2023). Toxic pollution of the environment reduces soil microbial diversity, lowers agro-product quality and food safety, and leads to increasing risks for farmers. Moreover, increasing resistance of the pathogens lowers the efficiency of chemically synthesized pesticides as a consequence of long-term application (Nicolopoulo-Stamati et al., 2016).

In order to favor a more sustainable agriculture in Vietnam, application of chemical fertilizers and pesticides were considered with growing concern by the government (Dao et al., 2015), and their substitution by environmentally friendly alternatives, such as the use of biologicals combined with “Good Agriculture Practices” (VietGAP), is a necessity for keeping the high-quality standard of Vietnamese agricultural products (Hoang, 2020).

Due to the problematic ecological as well as health hazardous properties of many of the chemically synthesized pesticides in use, new sustainable biological plant protection products are urgently needed. These environmentally friendly biocontrol agents should be specifically act against plant pathogens and effectively help to control common plant diseases without the environmentally harmful use of the chemically synthesized pesticides. We hypothesize, that plant growth promoting endophytic bacteria from the natural microbiome of healthy crops grown in pathogen-infested environments could help and serve as a basis for the development of improved biological pesticides.

This assumption was supported by a recent study in which we isolated endospore-forming Gram-positive bacteria with putative biocontrol function from healthy Vietnamese crop plants grown in fields infested with plant pathogens (Tam et al., 2020). Based on their draft genome sequences three main taxonomic groups able to control plant pathogens were distinguished: *Brevibacillus* spp. (Jähne et al., 2023), *Bacillus cereus* sensu lato, and 30 isolates assigned to the *B. subtilis* species complex (Fritze, 2004). Most of the latter group were representatives of *Bacillus velezensis* (Ruiz-García et al., 2005), a species which is known for its high potential to stimulate plant growth, and to suppress plant pathogens (Dunlap, 2019).

This study aims to perform a suitable approach for developing novel bioagents for disease control, which proved as being highly efficient in large field trials. Our approach includes the comprehensive analysis of (i) the genome sequences of the 30 isolates belonging to the *B. subtilis* species complex, (ii) their antagonistic metabolites, (iii) their action on selected plant pathogens, and (iv) their stimulatory effect on plant growth *in vitro* and *in vivo*. We show that bioformulations prepared with the most promising *B. velezensis* isolates, TL7 and S1, could be successfully applied in large field trials to protect black pepper and Arabica coffee plantations.

## 2 Materials and methods

### 2.1 Strain isolation, growth conditions and DNA isolation

Our strain isolation approach followed the procedure described by Wang and Liang (2014). The plant-associated bacteria were isolated from the rhizosphere soil and different organs of healthy crop plants, such as coffee, pepper, and others from fields located at Dak Nong, Dak Lac provinces, Vietnam, and infested with plant pathogens such as *Phytophthora palmivora*, *Fusarium oxysporum*, and the nematode *Meloidogyne* sp. (Tam et al., 2020). An overview about the isolation sources was given in **Supplementary Table S1**, and also reported previously (Tam et al., 2020). Routinely, leaf, stem and root of healthy plants were selected and taken to the laboratory for further processing. The plant organs were cut into 5 x 5 mm pieces, and washed twice with sterile water. Afterwards, the plant parts were dipped into 75% ethanol for one min, and then into 0.1% mercury dichloride (HgCl_2_) for two min. Then, the cuts were three times washed with sterile water, and transferred into 10 mL sterile water. The suspension was grounded in a sterile and chilled mortar. After 30 min. incubation, 0.1 mL of the solution was transferred to Luria broth (LB) agar plates, and allowed to grow for 72 h at 28°C. Finally, single colonies were purified, transferred to 50 mL fresh LB medium, and cultured in a shaker at 28°C, and 180 rpm for 24 h. In addition, a similar procedure was used for strain isolation from soil adhering at the roots of healthy plants. Before further processing, the soil containing suspension was incubated for 10 min. at 80°C. The purified strains were maintained as glycerol stocks (20 %, w/v) at −80°C. Cultivation of the bacterial strains and DNA isolation have been previously described (Tam et al., 2020; Blumenscheit et al., 2022).

### 2.2 Genome sequencing, assembly and annotation

Short-read sequencing was conducted in LGC Genomics (Berlin, Germany) using Illumina HiSeqq in a paired 150 bp manner as described previously (Blumenscheit et al., 2022). Long read sequencing was done in house with the Oxford Nanopore MinION with the flowcell (R9.4.1) and prepared with the Ligation Sequencing Kit (SQK-LSK109). *De-novo* assemblies were generated by using the hybrid-assembler Unicycler (https://github.com/rrwick/Unicycler v0.4.8, (Chen et al., 2018). The quality of assemblies was assessed by determining the ratio of falsely trimmed protein by using Ideel (https://github.com/phiweger/ideel). Genome coverage of the obtained contigs was 50 x in average.

Automatic genome annotation was performed using the NCBI Genome Automatic Annotation Pipeline (PGAP6.2) for the general genome annotation provided by NCBI RefSeq. Functional annotation was done by using the COG- (Tatusov et al., 2000), and the KEGG database (Kanehisa et al., 2023). Prediction of core and pan genomes, and comparative analyses were performed with the EDGAR3.0 pipeline (Dieckmann et al., 2021). Genomic islands (GI) were predicted with the webserver IslandViewer 4 (http://www.pathogenomics.sfu.ca/islandviewer/, Bertelli et al., 2017). Circular plots of genome and plasmid sequences were visualized with BioCircos (Cui et al., 2016).

Gene clusters for secondary metabolite synthesis were identified using the antiSMASH pipeline version 6 (Blin et al., 2021) under settings of all features, and BAGEL4 (van Heel et al., 2018). All biosynthetic gene clusters (BGCs) were investigated for their presence in the MIBiG repository (Kautsar et al., 2020).

### 2.3 Phylogeny and genome similarity assessment

The genome sequence data were uploaded to the Type (Strain) Genome Server (TYGS) available at https://tygs.dsmz.de (Meier-Kolthoff and Göker, 2019). Information on nomenclature was provided by the List of Prokaryotic names with Standing in Nomenclature (LPSN, available at https://lpsn.dsmz.de) (Meier-Kolthoff et al., 2022). The genomes were compared with all type strain genomes available in the TYGS database *via* the MASH algorithm (Ondov et al., 2016), and the ten strains with the smallest MASH distances were chosen per user genome. Using the Genome BLAST Distance Phylogeny approach (GBDP) the ten closest type strain genomes for each of the user genomes were calculated.

*In silico* DNA-DNA hybridization (dDDH) values were calculated in the TYGS platform using formula d_4_, which is the sum of all identities found in the high score segment pairs (HSPs) divided by the total length of all HSPs. Pan-genome analysis was performed using the EDGAR software package (Dieckmann et al., 2021). ANIb values were obtained with the Jspecies WS online service (https://jspecies.ribohost.com/jspeciesws/#anib). Species cut off: 95% (ANIb), 70% (dDDH); subspecies cut off: 97% (ANIb), 79% (dDDH).

The EDGAR3.0 pipeline (Dieckmann et al., 2021) was used for elucidating taxonomic relationships based on genome sequences. High throughput ANI analysis (FastANI) was performed according to Jain et al. (2018). To construct a phylogenetic tree for a project, the core genes of these genomes were computed. In a following step, alignments of each core gene set are generated using MUSCLE. Then the alignments were concatenated to one huge alignment. This alignment is the input for the FastTree software (http://www.microbesonline.org/fasttree/) to generate approximately-maximum-likelihood phylogenetic trees. The values at the branches of FastTree trees are not bootstrapping values, but local support values computed by FastTree using the Shimodaira-Hasegawa test.

### 2.4 Mass-spectrometric detection of bioactive peptides

Cultivation of organisms, sample preparation and mass spectrometric detection of the bioactive compounds produced by the investigated endophytes were essentially performed as described in (Mülner et al., 2020). The strains were grown on Landy agar plates (Landy et al., 1948) after incubation for 24 h at 30°C. The cells were then transferred into 20 µL of water and mixed with 80 µL of trifluoroacetic acid (TFA, Sigma-Aldrich, Deisenhofen, Germany). The suspension was incubated for 30 min at room temperature. Then 10 µL were diluted 1:10 with double-distilled water to achieve a cell extract with a final concentration of 8% TFA. For mass-spectrometric detection of the peptides, a Bruker Autoflex Speed TOF/TOF mass spectrometer (Bruker Daltonik, Bremen, Germany) was used with smart-beam laser technology using a 1 kHz frequency-tripled Nd-YAG laser. Two µL samples were mixed with 2 µL matrix solution (a saturated solution of a α-hydroxycinnamic acid in 50% aqueous acetonitrile containing 0.1% TFA), spotted on the target, air dried and measured. Mass spectra were taken by positive-ion detection in reflector mode. Monoisotopic masses were obtained with a resolution of 10.000.

### 2.5 Biocontrol activity against plant pathogens and plant growth promotion

Antifungal activity of the isolates was determined according to a method used by Soliman et al. (2022). Plugs (5 mm in diameter) with the pathogenic fungi were placed onto potato dextrose agar (PDA). Then, paper discs, saturated with the growing test bacteria (O.D._600 nm_ around 1.0), were added at a distance of 20 mm from the fungi. The cultures were incubated for six days at 27°C, and daily examined for colony diameter. Inhibition of fungal growth in vicinity of the bacteria was indicative for their antifungal activity. For the quantitative assay, two agar plugs (5 mm) containing either *Phytophthora palmivora* or *Fusarium oxysporum* were placed symmetrically onto potato dextrose agar (PDA). Then, a growing bacterial culture was streaked vertically between both plugs. The agar plates were incubated for seven days at 28°C. Then, the diameter of the fungal colonies was recorded. The experiments were repeated three times with ten replicates for each tested bacterial strain.

The bioassay of nematicidal activity was performed with *Caenorhabditis elegans* N2 (Carolina, U.S.A., https://www.carolina.com) fed with *Escherichia coli* OP50 cells. Culture and synchronization of the worms was performed as previously described (Lewis and Fleming, 1995). The L4 stage was used for two different bioassays performed as described previously (Liu et al., 2013). In the slow killing assay, around 40 L4 *C. elegans* roundworms were added to the nematode growth medium (NGM) agar plate containing the test bacteria. The mixture was incubated for 3-5 days at 25°C and daily inspected. In the liquid fast killing assay, the test bacteria were grown overnight under shaking (200 rpm) at 37°C in 3 mL liquid assay medium. 100µL of the bacterial culture were diluted with 500 µL M9 medium, and transferred into 12 well plates. Each well was seeded with 40 – 60 L4 stage N2 nematodes and the assay was performed at 25°C for 24 hrs. Mortalities of nematodes were defined as the ratio of dead nematodes to the total number of tested nematodes. All experiments were performed at least three times with each experiment comprising at least three replicates (N≥3).

An assay of plant growth was performed with wild type *Arabidopsis thaliana* (EDVOTEK, USA https://www.edvotek.com/) according to Budiharjo et al. (2014). The surface sterilized seeds were pre-germinated on Petri dishes containing half-strength Murashige-Skoog medium semi-solidified with 0.6% agar and incubated at 22°C under long day light conditions (16 h light/8 h dark) for seven days. Then, the roots of *Arabidopsis* seedlings were dipped into a diluted spore suspension of the test bacteria (10^5^ CFU ml^-1^) for five min. and five seedlings were transferred into a square Petri dish containing half-strength MS-medium solidified with 1% agar. The square Petri dishes were incubated in a growth chamber at 22°C at a daily photoperiod of 14 h. Fresh weight of the plants was measured 21 days after transplanting for estimating the ability of bacterial strains for growth promotion. Three replications per every variant including the control without bacterial treatment were performed.

The ´in planta´ assay using tomato plants infested with a natural isolate of *Meloidogyne* sp. was performed as following: The root-knot nematode *Meloidogyne* sp. was isolated from roots of infested pepper plants according to Hooper et al. (2005). Tomato plantlets were grown in pots containing natural soil, and exposed to the local subtropical climate conditions in the greenhouse of the PPRI, Hanoi. Test bacteria and second stage juveniles (J2) nematodes were added to the pots two weeks after transplanting. Ten weeks after infesting with the nematodes the number of knots in tomato plants was counted (Bridge and Page, 1980).

In all cases, at least three replications were performed. The negative controls were performed as described for the test bacteria, but without treatment with the test bacteria. More details, such as number of repetitions, calculation of the results, and statistical analyses, are given in in the **Supplementary Tables** referred to in the Results section.

### 2.6 Greenhouse and field trials

#### 2.6.1 Bioformulations manufactured from TL7 and S1

Bioformulations, named ENDOBICA1 and BIORHIZO1, were prepared from *B. velezensis* TL7 and *B. velezensis* S1, respectively. Bacterial cells were cultivated under shaking (220 rpm/min.) in LB supplemented with Mg^++^ ions for at least two days at 28°C. Further processing was performed, such as adjusting the concentration of the culture liquid to a titer of 3.5 x 10^10^ cells mL^-1^, and adding of adjuvants and other additives for stabilization. Application of ENDOBICA1 plant took place as a water-diluted spray directed to the leaves and the stem. Diluted BIORHIZO1 was applied directly to the soil in vicinity of the plant roots.

#### 2.6.2 Greenhouse

The “greenhouse” (location: PPRI, Hanoi), means here the so called “net”-house type, which is typical in tropical and subtropical regions. Here, the roof and the side walls are covered with nets not with glass panes. Plants grown in the net-house, therefore were protected from insects but directly exposed to natural subtropical climate conditions. Only watering was regularly performed.

All experiments were performed with completely randomized design with at least three independent repetitions.

Black pepper and coffee trees (variety Robusta) from the nursery were grown for two years in pots containing either 5kg (black pepper plants) or 10 kg (Robusta coffee trees) of the local Red River alluvial soil with a color ranging from bright brown to purple brown. In case of black pepper plants, 0.2 g mineral NPK (N 20: P 20: K 15) fertilizer, and 400 g earthworm excrements mixed with 100 g of rice husks were added to each pot. Before planting, the soil was sterilized by autoclaving at 120°C for 45 min. Two months after planting a second dose (0.1 g) of mineral NPK fertilizer was added to each pot. The same ingredients were added in proportional amount to the pots containing 10 kg soil and the planted Robusta coffee trees. 200 mL of the 5% liquid bioformulation corresponding to 7 x 10^10^ cfu/plant were poured to each pot. One month after this treatment, 2000 individuals of the pathogenic J2 *Meloidogyne* sp. nematode were added to each pot. Finally, two months after the treatment with the beneficial bioformulation, the plants were inoculated with either the pathogenic oomycete *Phytophthora palmivora* or the pathogenic fungus *Fusarium oxysporum* yielding a final concentration of 10^5^ cfu g^-1^ soil, respectively. The negative controls were performed in the same way, but 200 mL tap water were used for treatment instead of the diluted bioformulations. Every treatment was carried out on ten plants. The results were recorded six months after the treatment with the bioformulations. The parameters for measuring the plant growth promoting effect on pathogen-infested coffee and black pepper plants were: height, plant canopy diameter, number of shoot branches, and the leaf chlorophyll content index (SPAD values, measured with the Chlorophyll Meter SPAD502Plus, Konica Minolta). The assays were performed as described by Nguyen et al. (2021).

#### 2.6.3 Field trials

The field trials in which the bioformulations were used for the treatment of coffee trees and black pepper plants (Vinh Linh variety) were performed in large experimental plots with a size of one hectare at two different locations in Viet Nam. Each of the one-hectare plots including the control without the bioformulations was fertilized with a mixture of 5 tons humic fertilizer, and 50 kg mineral NPK fertilizer (N 20: P20: K15). Harvesting was performed after six months when the color of the fruits was changed to red. The harvest yield was determined by estimating the total weight of the dried coffee beans or the dried pepper fruits, respectively. For data collection obtained of an area of 10,000 m^2^ per trial, five selected areas with 20 plants each were selected for each-one ha plot according to a fixed scheme developed by PPRI (**Supplementary Figure S1**).

The trials with the Arabica coffee trees were performed in the mountain region of Cau Dat, Xuan Truong, Da Lat, Lam Dong, Viet Nam (11°50’11.0”N 108°32’29.9”E) at an altitude of 1,500 -1,600 m above sea level. The basalt containing soil is red colored, and rich on clay minerals, well drained and fertile. The soil pH measured in H_2_O is 5.5 to 6. The average temperature ranges from 18 to 21°C. The highest temperature is not exceeding 30°C, and the lowest temperature is not less than 5°C. Da Lat has two distinct seasons: the rainy season and the dry season. Dry season: from November to April, coinciding with the northeast monsoon season. The weather is generally warm, sunny, less cloudy, no rain, low temperature at night. Occasionally there will be rain in the afternoon, sometimes hail. The rainy season is from May to October, coinciding with the southwest monsoon season, often with heavy or prolonged rain. The average annual rainfall is 1562 mm. The humidity is 82%. For treatment of one ha with 6,250 Arabica coffee trees, 12.5 L of the bioformulation (3.5 x 10^10^ cfu mL^-1^) were used, and diluted 1:1000 into water immediately before use. The control was performed with 2 L water per coffee tree instead treatment with the bioformulation.

The field trials with black pepper plants were performed under tropical monsoon climate conditions in the Chu Se, Gia Lai, Viet Nam (13°39’27.6”N 108°06’42.6”E) region at an altitude of 700 – 800 m above sea level. The area is flat or slightly sloping. The basalt containing soil is red colored, and rich on clay minerals, well drained and fertile. The soil pH measured in H_2_O is 5.5 to 6. The climate is characterized by abundant humidity and high rainfall. The rainy season usually starts from May and ends in October. The dry season from November to April next year. The annual average temperature is 22 – 25^°^C. The average annual rainfall amounts to 2,200 - 2,500 mm. Gia Lai’s climate and soil are very suitable for the development of many short- and long-term industrial crops. For treatment of one ha with 1,600 black pepper “pillars” (trellis) with one to three plants, each, 3.2 L of the bioformulation (3.5 x 10^10^ cfu mL^-1^) were used, and diluted 1:1000 into water immediately before use (corresponding to 2 L per pillar).

### 2.7 Tests for estimation of plant pathogens

The number of zoospores of *P. palmivora* in soil was estimated six months after the treatment with the bioformulations by counting the number of zoospores able to “bait” and to decolorize rose petals (Drenth and Sendall, 2001). 100 g of dried root adhering soil were sieved through a 2-mm mesh, and suspended in 200 mL distilled water by stirring with a glass rod. After incubation overnight, 100 squared pieces of rose petals (1 mm x 1 mm, “traps”) were added to the suspension. After one to two days, the decolorized petals were examined by light microscopy for the presence of zoospores, and the number of decolorized petals containing zoospores were counted.

The number of *F. oxysporum* var. *coffea* colonies g^-1^ soil were estimated six months after treatment with the bioformulation by plating of 10^-4^ diluted soil samples on PDA (potato dextrose agar) and CLA (green rice stem agar), and incubating at 28°C for one week according to Burgess et al. (2008). The *Fusarium oxysporum* f. *coffea* prototype occurring in the infested crop field soil, and in infested coffee plant roots, was previously isolated according to Burgess et al., 2008, and taxonomically assigned as representative of the *F. oxysporum* species by 18S rRNA sequencing. Interestingly, its 18S rRNA sequence was found identical with the sequence (OP010081.1) from *F. oxysporum* ZEHFO from Saudi-Arabia, reported as being associated with the wilt disease of Coffee arabica. Its pathogenicity according to Koch´s postulate was proven against coffee trees, which were damaged after treatment with the *F. oxysporum* isolates. Only colonies appearing white cottony and with the typical dark-purple undersurface pigment after growth in PDA, supplemented with streptomycin sulfate (1g/L) and neomycin sulfate (0.12 g/L), were counted. In addition, the morphological features typical for *F. oxysporum* (Burgess et al., 2008) were identified by light microscopy performed with selected colonies grown on carnation leaf or green rice stem agar.

The density of nematodes in soil was estimated six months after treatment with the bioformulations by counting the number of nematodes detected by light microscopy in a defined amount of soil (100 g) according to Hooper et al. (2005). The number of nematodes in plant roots was estimated according to Hooper et al. (2005). The washed and surface disinfected roots were separated from the stem at the soil line, and the number of nematodes in the cut root pieces were counted.

The disease severity index (%) for yellow leaf and root rot (YLRR) disease on coffee trees was calculated as described (Townsend and Heuberger, 1943) according to the formula:


$$Index\;of\;disease\;severity\left(\%\right)=\frac{\sum \left(\alpha \times b\right)}{N\times T}\times 100$$

where:

✔ ∑(axb) : Sum of the product of the number of infected plants and its respective level of disease.
✔ T: The highest level of disease.
✔ N: Total number of investigated plants.

For YLRR disease on coffee plants, there are five levels of disease:

Level 0: plant is healthy or not infected.
Level 1: the percentage of yellowing leaves is ≤25%.
Level 2: the percentage of yellowing leaves is between 25% to 50%. The lateral roots and the main root are in part knotted or black rotted. Plant growth is impaired.
Level 3: the percentage of yellowing leaves is between 50% to 75%. Majority of the lateral roots and the main root are knotted or black rotted. Plant growth is heavily impaired.
Level 4: the percentage of yellowing leaves is ≥75%. Plants start to die.

The incidence rate of fast death disease (%) in black pepper plants is defined as:


$$Incidence\left(\%\right)=\frac{A}{B}x100$$

where:

A: the number of black pepper plant infected with fast death disease.
B: Number of all investigated black pepper plants

The greenhouse experiments for determining the number of plant pathogens in soil and severity and incidence of the YLRR and fast death disease were performed with ten plants and in three repetitions. The control plants were not treated with the bioformulation. Examples about calculating occurrence of plant pathogens, and severity and incidence of disease are given in **Supplementary Tables S12**, **S13** for Robusta coffee and **Supplementary Tables S16**, **S17** for black pepper. Also here, the greenhouse experiments were performed with ten plants and three repetitions. The details of the corresponding field trials were described in **Supplementary Tables S14**, **S15** (Arabica coffee), and **Supplementary Tables S18**, **S19** (black pepper).

### 2.8 Data analysis

Except large-scale field trials, the data obtained from biocontrol and plant growth promotion experiments were analyzed using one-factorial analysis of variance (ANOVA). Mean values were calculated from the results of the replicates (n≥3). The Fisher´s least significant difference (LSD) test was conducted as *post-hoc* test for estimating significant differences (p≤ 0.05) between the mean values.

The **formula** for the least significant difference is:


$$LSD_{A,B}=t_{0.05/2,DFW}\sqrt{MSW(1/n_{A}+1/n_{B})}$$

Where:

t = critical value from the t-distribution table
MSW = mean square within, obtained from the results of the ANOVA test
n = number of scores used to calculate the means.

Every experiment was conducted using a completely randomized design.

## 3 Results

### 3.1 Molecular taxonomy and comparative genome analysis of 30 isolates from Vietnamese crop plants representing the *B. subtilis* species complex

#### 3.1.1 Molecular taxonomy revealed that the isolates belong to the *Bacillus subtilis* species complex

**Figure 1:**
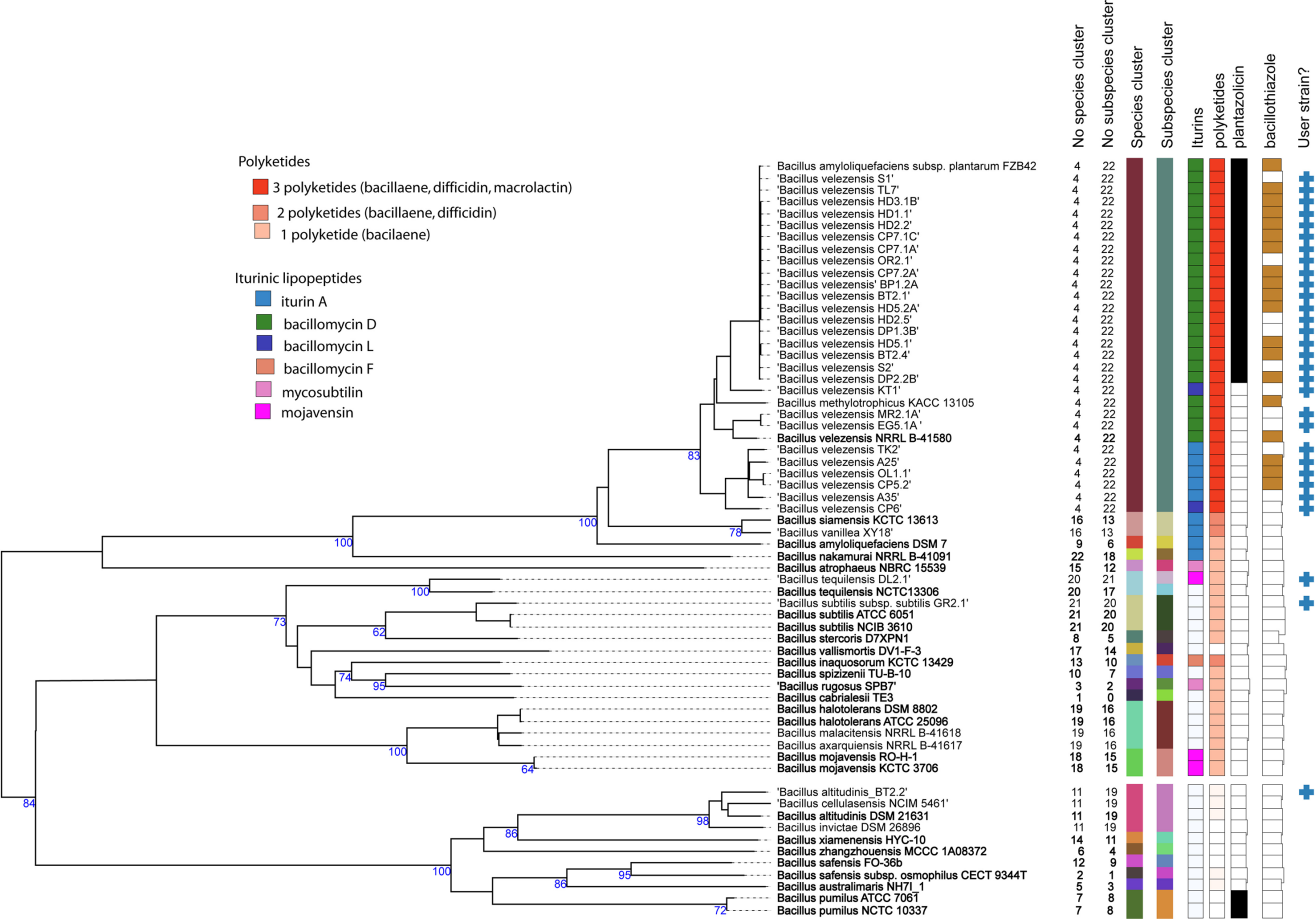
Phylogenetic tree inferred with FastMe 2.1.6.1 (Lefort et al., 2015) from GBDP distances calculated from whole genome sequences using the Type (Strain) Genome Server TYGS (https://tygs.dsmz.de). Analysis was performed using both, Maximum Likelihood and Maximum Parsimony, with 31 type strains (labelled by bold letters) and 30 genome sequences obtained from the *Bacillus* strains isolated from Vietnamese crop plants (labelled by the blue crosses at the right). The numbers above branches are GBDP pseudo-bootstrap support values > 60% from replications, with an average branch support of 85.1%. The first two colored columns to the right of each name refer to the genome-based species and subspecies clusters, as determined by dDDH cut-off of 70 and 79%, respectively. *B*. *tequilensis* DL2.1 represents a subspecies different from that of the type strain NCTC 13306. The clustering yielded 22 species clusters and the provided query strains were assigned to four of these. The third column refers to the iturinic lipopeptide, and the fourth column to the polyketide clusters detected in the genome sequences. The fifth column refers to the occurrence of plantazolicin, and the sixth column to the occurrence of bacillothizole gene clusters. The tree was rooted at the midpoint.

Since16S rRNA sequences are often not sufficient for species discrimination, we used the genome sequences for taxonomical assignment. The phylogenetic tree (**Figure 1**), containing the genomes of the 30 isolates from Vietnamese crop plants and of numerous type strains belonging to the *B. subtilis* species complex, was constructed with the Type (Strain) Genome Server TYGS (Meier-Kolthoff and Göker, 2019). The isolates were distributed in four different clusters, representing the species *B. velezensis*, *B. tequilensis*, *B. subtilis*, and *B. altitudinis*. Within the *B. velezensis* cluster, which contained 27 isolates, three subclusters can be distinguished. Eighteen isolates including TL7 and S1 formed a subcluster closely related with FZB42 (Borriss et al., 2011). Isolates MR2.1A and EG5.1A formed together with the type strain *B. velezensis* NRLL B-41580 (Ruiz-García et al., 2005) a second subcluster. A third branch consisted of five isolates. However, when compared with the type strain NRLL B-41580, their ANIb - and dDDH values were found above the species and subspecies cut off (**Supplementary Table S1**; **Supplementary Figure S5**), suggesting that they belong to one subspecies and no further discrimination according to their taxonomic level was necessary.

The isolate DL2.1, which was forming a species cluster together with *B. tequilensis* NCTC 13306 (Gatson et al., 2006), showed values below the subspecies cut off when compared with the type strain NCTCC 13306 (ANIb: 96.89%, dDDH:78.5%), suggesting that the isolate DL2.1 represented a novel subspecies.

#### 3.1.2 Pan genome analysis of the *Bacillus velezensis* isolates

The functional category analyses of the 27 *B. velezensis* isolates with *B. velezensis* FZB42 as reference revealed 110,824 COG functional categories distributed in core, dispensable, and single genes, A relatively high percentage of around 11% was dedicated to carbohydrate transport and metabolism. Nearly 5% were predicted to be involved in synthesis of secondary metabolites (**Supplementary Figure S6**). No differences between strains isolated from surface-sterilized plant material (roots, stems, and leaves), and strains isolated from the plant rhizosphere were detected (**Supplementary Figure S7**).

Singletons were defined as unique genes, not occurring in the other *B. velezensis* strains used for comparison. The majority of singletons were phage genes, and genes involved in synthesis of restriction-modification systems, ComX- pheromones, plasmid replication and mobilization, lantibiotics and other ribosomally synthesized and post-translationally modified peptides (RiPPs). The number of singletons detected in the isolates were obviously not dependent on their life style, and a direct comparison between the endophytic isolates, and the isolates obtained from the rhizosphere yielded no genes specifically connected with the endophytic life style. The strain with the highest number of singletons (210) was *B. velezensis* TK2, a strain isolated from plant rhizosphere (**Supplementary Table S7**).

#### 3.1.3 Whole genome analysis of *Bacillus velezensis* BP1.2A and BT2.4 revealed their close similarity with FZB42

In order to characterize the *B. velezensis* subcluster 1, sharing high similarity with FZB42 (**Supplementary Table S1**), in more detail, two randomly selected isolates, BP1.2A and BT2.4, were chosen for further analysis. Both strains were fully sequenced using a combined approach of two sequencing technologies which generated short paired-end reads obtained with Illumina HiSeq and long reads obtained with the Oxford nanopore MinION sequencing technology. The obtained sequences were then used for hybrid assembly (Blumenscheit et al., 2022). The data describing their general genomic features, summarized in **Supplementary Table S2**, have been already listed in a recent data paper (Blumenscheit et al., 2022), but were not comprehensively discussed until now.

**Figure 2:**
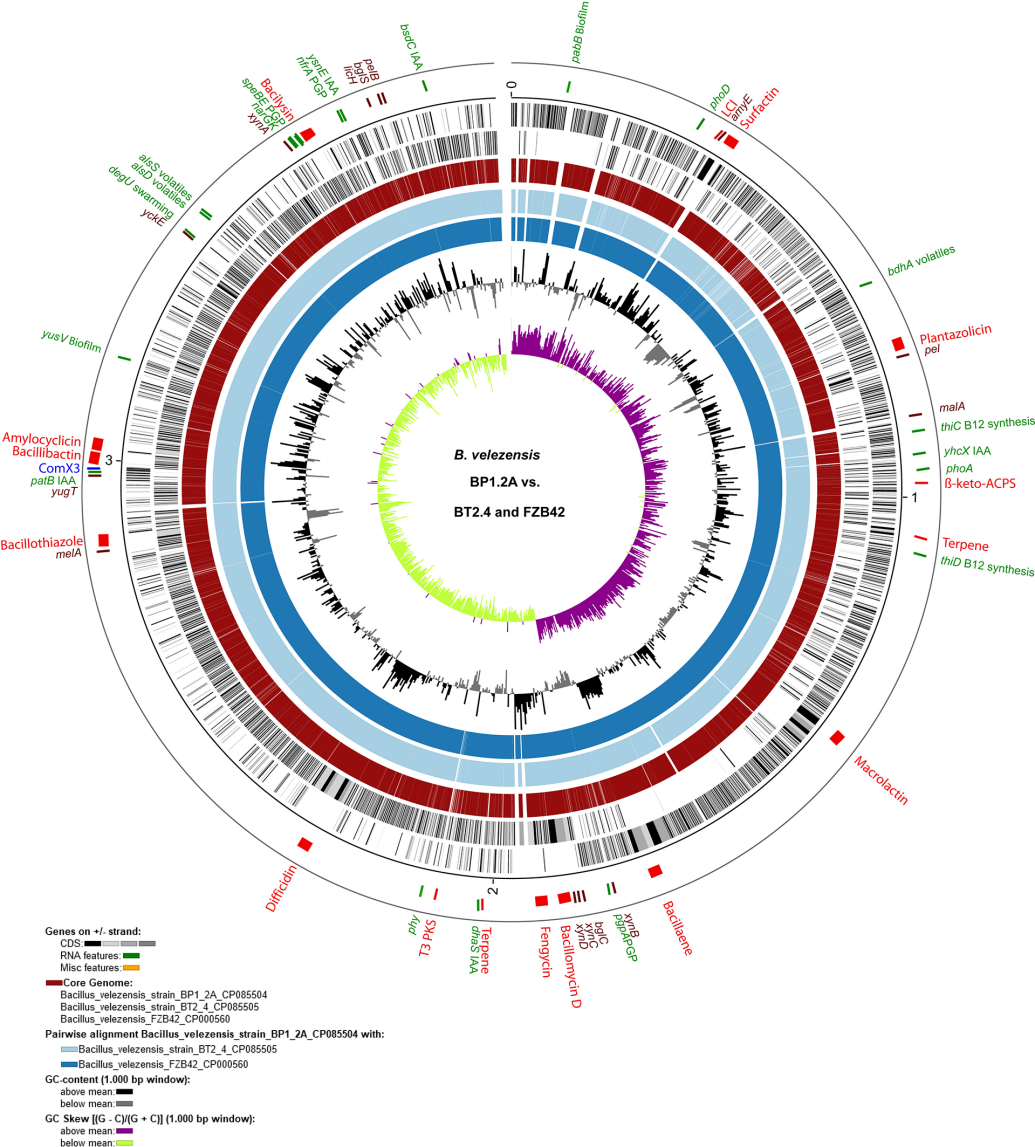
Circular plot of the BP1.2A genome (CP085504) compared with BT2.1.4 and FZB42. The plot was generated with BioCircos embedded within the EDGAR3.0 package (Dieckmann et al., 2021). The outer circle shows the position of the 17 BGCs (red), the genes and gene clusters involved in plant growth promotion (green), and of the enzymes degrading carbohydrate macromolecules (dark brown). Homologous regions representing the core genome formed by the three strains are shown in the fourth circle. Circles 5 and 6 represent the pairwise alignments with BT2.4 and FZB42, respectively. The inner circles 7 and 8 represent the GC-content, and the GC-skew [(G-C)/(G+C)] within 1,000 bp windows. Genes involved in plant growth promotion and degradation of plant macromolecules are listed in **Supplementary Table S5**.

Despite, that both strains were isolated from different sources (**Supplementary Table S1**), sequences of both strains were found closely related to *B. velezensis* FZB42, the model strain for Gram-positive, plant-beneficial bacteria, which has been isolated from a very remote area, an infested sugar beet field in Germany (Fan et al., 2017). The Venn diagram (**Supplementary Figure S2**) showed that the core genome of the three strains harbored a total of 3,633 genes (**Supplementary Table S3**). Only 75 genes of FZB42 were not detected in strains BP1.2A, and BT2.4. By contrast, 46 genes detected in BP1.2A and BT2.4 did not occur in FZB42. The pan genome formed by the three strains consisted of 3,757 genes (**Supplementary Table S4**). The chromosome of the isolate *B. velezensis* BP1.2A was used as reference for computing the core genome against *B. velezensis* BT2.4 and *B. velezensis* FZB42 (**Figure 2**). Genes probably involved in plant growth promotion, degradation of plant macromolecules (**Supplementary Table S5**), and synthesis of secondary metabolites, are indicated in the circular plot.

#### 3.1.4 Biosynthetic gene clusters encoding secondary metabolites

**Figure 3:**
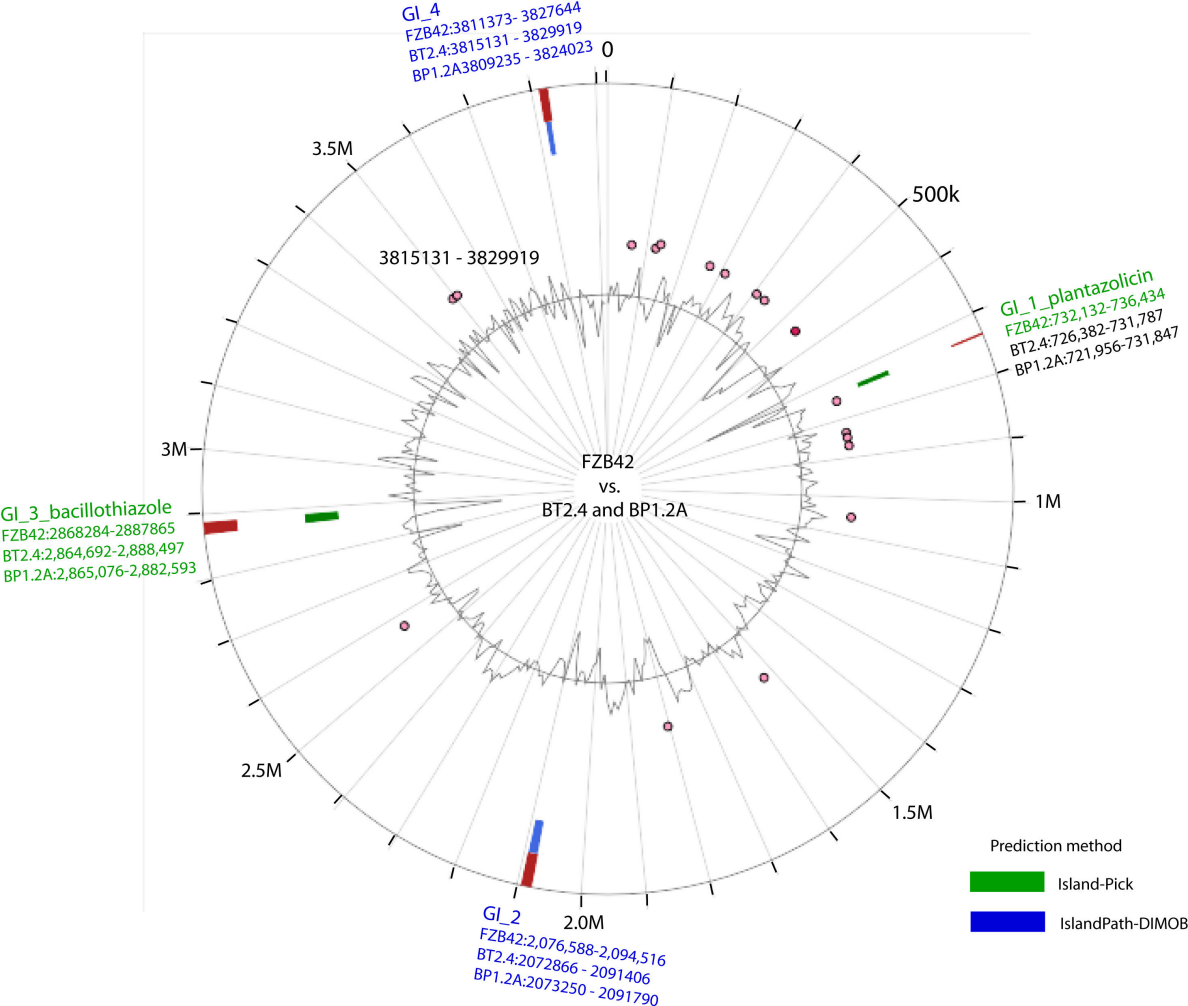
Occurrence of the genomic islands (GIs) in the chromosomes of *B*. *velezensis* BP1.2A, and *B*. *velezensis* BT2.4 using IslandViewer 4 (Bertelli et al., 2017). *B*. *velezensis* FZB42 is shown for comparison. Predicted GIs are shown as blocks colored according to the prediction method; Island Pick (green), Island‐Path‐DIMOB (blue), as well as the integrated results (dark red). The grey line within the inner circle shows deviations of the average GC‐content. The genes present in the GIs are listed in **Supplementary Table S6**.

Genome mining using the software pipelines of antiSMASH and BAGEL4 was performed with the genomes of all the 30 isolates representing the *B. subtilis* species complex. Our survey yielded 17 gene clusters involved in biosynthesis of secondary metabolites in *B. velezensis*, which were found distributed within different parts of the genomes. **Table 1** gives an overview about their position in the genomes of BP1.2A, BT2.4, and, for comparison, FZB42. Two of them, plantazolicin (Scholz et al., 2011), and the recently described bacillothiazole (Shen et al., 2022), were found to be not generally conserved throughout the species *B. velezensis*, but occurred sporadically in genomic islands, probably acquired by horizontal gene transfer (**Figure 3**). The gene cluster involved in biosynthesis of plantazolicin was detected in the genomic island of FZB42 covering region 732,136 – 736,434. The plantazolicin gene clusters, occurring in BP1.2A, and BT2.4, were detected in the corresponding regions (**Supplementary Table S6**). The bacillothiazole gene cluster occurred in all three strains in the genomic islands, and was covering the regions 2,864,692 – 2,888,497 (BT2.4), 2,865,076 – 2,882,593 (BP1.2A), and 2,868,284 – 2,887,865 (FZB42), respectively.

**Table 1:** Detection of 17 gene clusters involved in synthesis of secondary metabolites (BGCs) using antiSMASH (Blin et al., 2021) and BAGEL4 (van Heel et al., 2018) in the genomes of *B. velezensis* BP1.2A (CP085504), and *B.velezensis* BT2.4 (CP085505).

Region	BP1.2A (CP085504.1)	BT2.4 (CP085505.1)	FZB42 (CP000560.2)	Similarity MIBIG/NCBI	
**LCI** (antimicrobial peptide)	296,346 - 316,483	296,348 - 316,483	300,862 - 320,997	100%	Bacillus BAGEL4
**Surfactin** (NRP, lipopeptide)	318,208 - 383,067	318,208 - 383,067	322,723 - 387,582	95%	BGC0000433
**Plantazolicin** 91.1 RiPP : LAP	717,159 - 740,336	717,099 - 740,276	721,674 744,851	100%	BGC0000569 BAGEL4
**ß-keto-ACPS** PKS-like	935,682 - 976,926	935,298 - 976,542	940,739 - 981,983	100%	Bacillus
**Terpene** Squalene/phytoene	1,062,552 - 1,079,781	1,062,168 - 1,079,397	1,074,783 - 1,075,523	100%	Bacillus
**Macrolactin H** Polyketide	1,366,841 - 1,453,226	1,366,457 - 1,452,842	1,371,897 - 1,458,282	100%	BGC0000181
**Bacillaene** polyketide + NRP	1,676,755 - 1,777,357	1,676,371 - 1,776,973	1,681,811 - 1,782,413	100%	BGC0001089
**Bacillomycin D** NRP + polyketide	1,866,123 - 1,903,373	1,865,739 - 1,902,989	1,871,179 - 1,908,429	100%	BGC0001090
**Fengycin** NRP	1,907,878 - 1,963,948	1,918,319 - 1,963,564	1,923,759 - 1,969,004	100%	BGC0001095
**Terpene** Sporulene	2,010,880 - 2,032,763	2,010,496 - 2,032,379	2,024,219 - 2,026,102	100%	Bacillus
**T3PKS** type III-PKS	2,099,249 - 2,140,349	2,098,865 - 2,139,965	2,102,588 - 2,143,688	100%	Bacillus
**Difficidin** polyketide	2,269,142 2,362,931	2,268,758 - 2,362,547	2,344,012 - 2,286,309	100%	BGC0000176
**Bacillothiazole** NRP	2,851,295 - 2,900,808	2,850,911 - 2,906,712	2,873,990 - 2,884,225	100%	BGC0002641
**ComX3** 320.1 pheromone	2,994,084 - 2,994,275	2,999,980 - 3,000,171	2,997,539 - 2,997,712	100%	*B. velezensis* BAGEL4
**Bacillibactin** NRP siderophore	3,017,800 - 3,024,927,	3,023,696 - 3,030,823	3,021,021 - 3,033,995	100%	BGC0000309
**Amylocyclicin** RiPP head to tail cycl.	3,039,655 - 3,045,228	3,045,551 - 3,051,124	3,043,470 - 3,049,481	100%	BGC0000616 BAGEL4
**Bacilysin** other	3,574,134 3,615,552	3,580,030 - 3,621,448	3,593,882 - 3,599,780	100%	BGC0001184

For comparison FZB42 (CP000560.2) was also analyzed. Similarity to known metabolites listed in the MIBiG 3.0 repository (Kautsar et al., 2020) is indicated.

**Figure 4:**
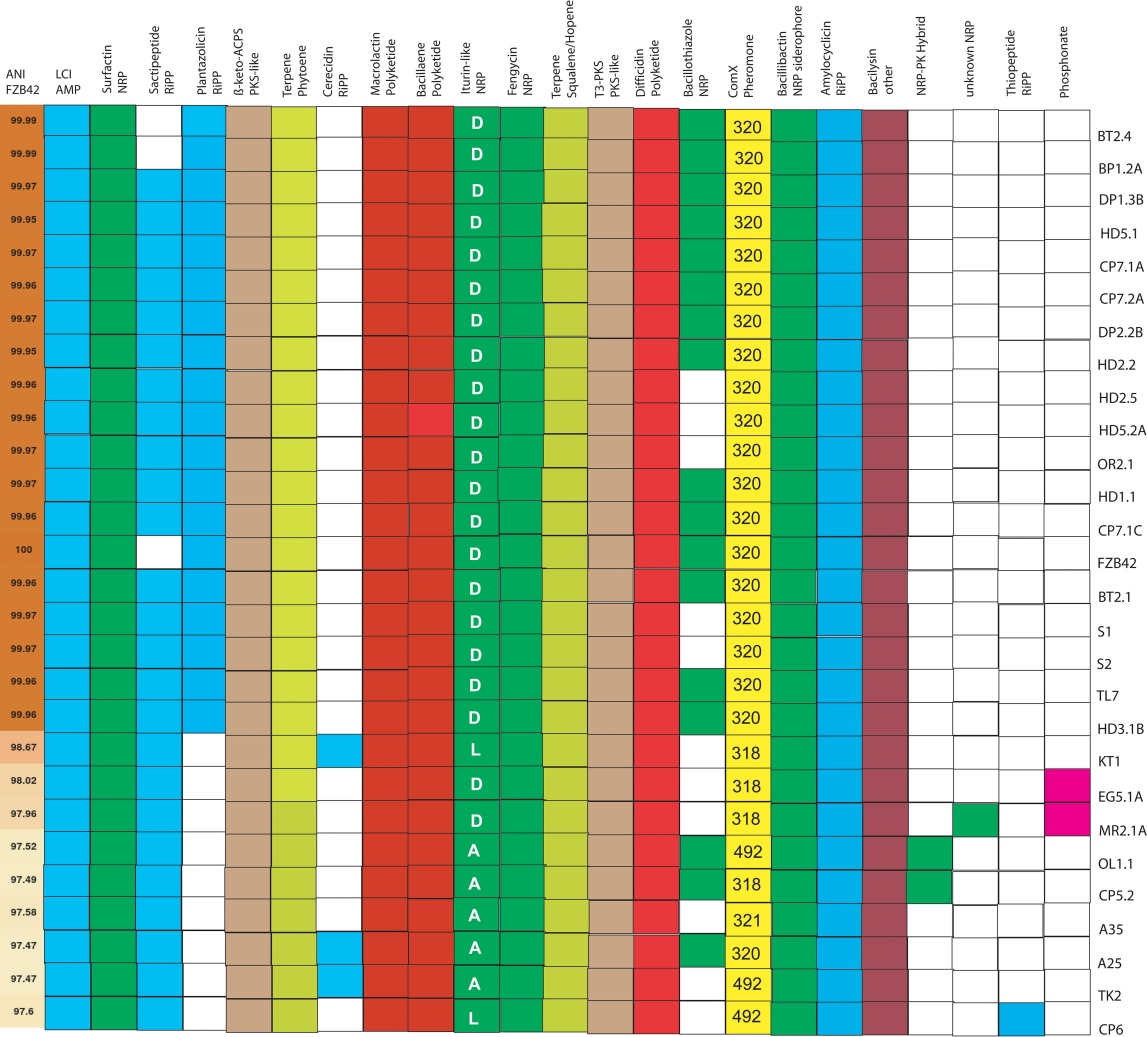
Occurrence of 23 biosynthetic gene clusters encoding secondary metabolites in 27 *B. velezensis* isolates obtained from Vietnamese crop plants. Representatives of cyclic iturinic lipopeptides were bacillomycin D (D), bacillomycin L (L), and iturin A (A). Gene clusters for synthesis of four different types of ComX pheromones (318, 320, 321, 492) were detected with BAGEL4. The first row shows the ANIb values of the *B*. *velezensis* genomes when compared with FZB42.

Fifteen “canonical” gene clusters were detected in the 27 *B. velezensis* isolates (**Figure 4**). They were responsible for non-ribosomal synthesis of the polyketides macrolactin, bacillaene, and difficidin, the lipopeptides surfactin, fengycin, and iturin-like compounds, the NRP-siderophore bacillibactin, and the dipeptide bacilysin. Biosynthesis of three different iturinic lipopeptides was predicted: The *B. velezensis* isolates, closely related to FZB42, harboured the gene cluster predicted to synthesize bacillomycin D (BGC0001090). Some of the more distantly related *B. velezensis* isolates harbored gene clusters predicted to be involved in the synthesis of iturin A (BGC0001098) and bacillomycin L, respectively (**Supplementary Figure S8**).

The gene clusters for ribosomal synthesis of RiPPs, such as LCS, and amylocyclicin were found in every *B. velezensis* isolate (**Supplementary Figure S9**). Well conserved in all *B. velezensis* isolates were also the clusters containing the genes for ß-keto-ACPS (PKS-like), and iterative type III polyketide synthesis, for synthesis of two different terpenes, and for synthesis of four different types of the ComX pheromone (**Supplementary Figure S10**). As expected, the occurrence of the gene clusters involved biosynthesis of the RiPP plantazolicin, and the NRP-bacillothiazoles varied within the *B. velezensis* isolates. The BGC for plantazolicin synthesis was only detected within the closest relatives of FZB42, whilst the BGCs for bacillothiazole appeared more scattered in different isolates.

Six additional gene clusters, not occurring in FZB42, BT2.4, and BP2.1A, were identified in other *B. velezensis* isolates. Two uncharacterized gene clusters involved in non-ribosomal synthesis of NRP, and NRP-PK hybrids were detected in *B. velezensis* OL1, CP5.2, and MR2.1A (**Supplementary Figure S11**).

Two uncharacterized biosynthetic gene clusters were detected in *B. altitudinis* BT2.2 (**Supplementary Figure S12**): The NRP-independent siderophore cluster contained genes with similarity to schizokinen (BGC0002683), and a non-ribosomal peptide gene cluster harbouring genes similar to the genes present in BGC0000381 responsible for synthesis of the surfactant lichenicidin in *B. licheniformis*.

RiPPs of the sactipeptide type were found in most *B. velezensis* strains, but did not occur in FZB42, BP2.1A. and BT2.4 (**Supplementary Figure S13**). BGCs predicted to synthesize different types of lanthipeptides were detected in *B. subtilis* and *B. velezensis* (**Supplementary Figure S14**). The subtilomycin A (lanthipeptide class I) gene cluster (BGC0000560) was detected in *B. subtilis* GR2. *B. velezensis* isolates harboured BGCs predicted to synthesize lanthipeptides representing class II (lichenicidin) and class IV (andalusin A). Other lanthipeptides, such as the thiopeptide thiocillin (*B. velezensis* CP6) were also detected (**Supplementary Figure S15**).

Known and hitherto unknown RiPPs were detected in *B. altitudinis* BT2.2. The head-to-tail cyclized pumilarin resembled amylocyclicin in *B. velezensis*. Another head-to-tail cyclized peptide (BhlA/UviB family) was similar to enterocin-48 from *Enterococcus lactis*. The leaderless class II bacteriocin aureocin A53 exhibited similarity to lacticin from *Lactococcus lactis* (**Supplementary Figure S16**). The BGCs responsible for synthesis of phosphonate were detected in *B. velezensis* EG5.1A, and MR2.1A (**Supplementary Table S8**).

### 3.2 Detection of bioactive peptides by MALDI-TOF mass spectrometry

**Figure 5:**
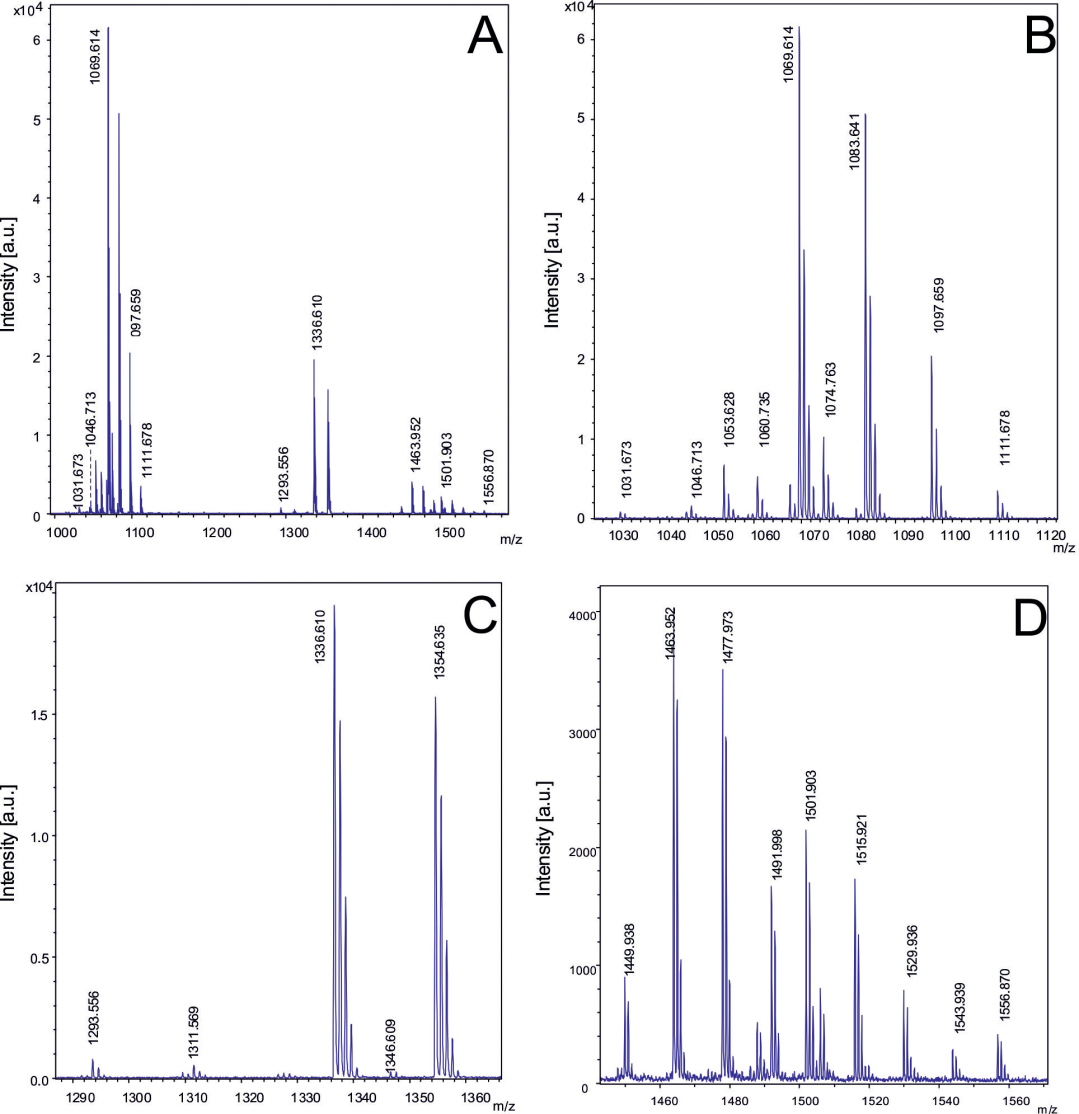
Detection of bioactive peptides produced by *B*. *velezensis* S1. **(A)** MALDI-TOF mass spectrum in the mass range from *m/z* = 1000 – 1500 of a cell-extract of strain S1 showing bacillomycin D species as the most prominent signals. Figures B, C, D show extended views of the signals visible in **Figure 6A**. **(B)**: Mass spectrum of C14-, C15- and C16 bacillomycin D, and C14 and C15-surfactin in the mass range from *m/z*=1000 to 1120. **(C)**: Mass spectrum of plantazolicin and its hydrated form with mass numbers of *m/z* = 1336.6 and 1354.6. **(D)**: Mass spectrum of the C14 - C17 fengycin species in the mass range *m/z* = 1400-1550. *B*. *velezensis* S1 was grown on Landy-agar for 48 (h) Cell material was picked from agar-plates, extracted with 50% ACN/0.1%% TFA, and processed as described under Materials and methods.

The mass-spectrometric detection of bioactive peptides revealed that the *B. velezensis* isolates were able to synthesize the lipopeptides of the iturin family, either bacillomycin D or iturin A, the fengycins, and surfactin (Koumoutsi et al., 2004), the siderophore bacillibactin (Chen et al., 2009b), and plantazolicin (Scholz et al., 2011, **Supplementary Table S8**). Cyclic lipopeptides produced by FZB42, and other representatives of the *B. velezensis* species are known for their strong action against plant-pathogenic fungi (Gu et al., 2017), and to enhance plant defense responses such as induced systemic response (ISR, Chowdhury et al., 2015). Plantazolicin has been reported as being able to suppress plant-pathogenic nematodes (Liu et al., 2013). Most members of the *B. velezensis* subcluster 1 produced detectable amounts of surfactin, bacillomycin D, and plantazolicin. **Figure 5** presents as a representative example of subcluster 1 the metabolic profile obtained from the isolate *B. velezensis* S1 grown on Landy agar, and further processed as described in Materials and Methods. Under these conditions, several bacillomycin D species containing lipopeptide chains of variable length, ranging from C14 to C16, were detected as the dominating compounds in surface extracts of S1.

The following bacillomycin D species were detected:

- C14-bacillomycin D: [M +H; Na;K]+ = 1031.7/1053.6/1069.6;
- C15-bacillomycin D: [M + H;Na;K]^+^ = 1045.7/1067.6/1083.6;
- C16- bacillomycin D: [M +H; Na;K]^+^ = 1059.7/1081.6/1097.7.

Surfactin was represented by:

- C14-surfactin: [M + Na : K]^+^ = 1044.7/1060.7:
- C15-surfactin: [M + Na : K]^+^ = 1058.7/1074.7.

The detected fengycins A and B were:

- C15-fengycin A: [M +H;Na;K]^+^= 1449.9/1471.9/1487.9;
- C16-fengycin A: [M +H;Na;K]^+^= 1463.9/1485.9/1501.9;
- C17-fengycin A: [M +H;Na;K]^+^= 1477.9/1499.7/1515.9;
- C16-fengycin B: [M + H,Na,K]^+^ = 1491.9/1513.9/1529.9.

Plantazolicin and its hydrated form were also detected:

- plantazolicin: [M + H]^+^ = 1336.6/1354.6.

Corresponding results were obtained with the other representatives of subcluster 1 including isolate TL7. It was speculated that variations in the fatty acid chain-length of the lipopeptides affect their biological activity (Ramachandran et al., 2017).

### 3.3 *Bacillus velezensis* strains are highly efficient in suppressing plant pathogens and promoting plant growth

**Figure 6:**
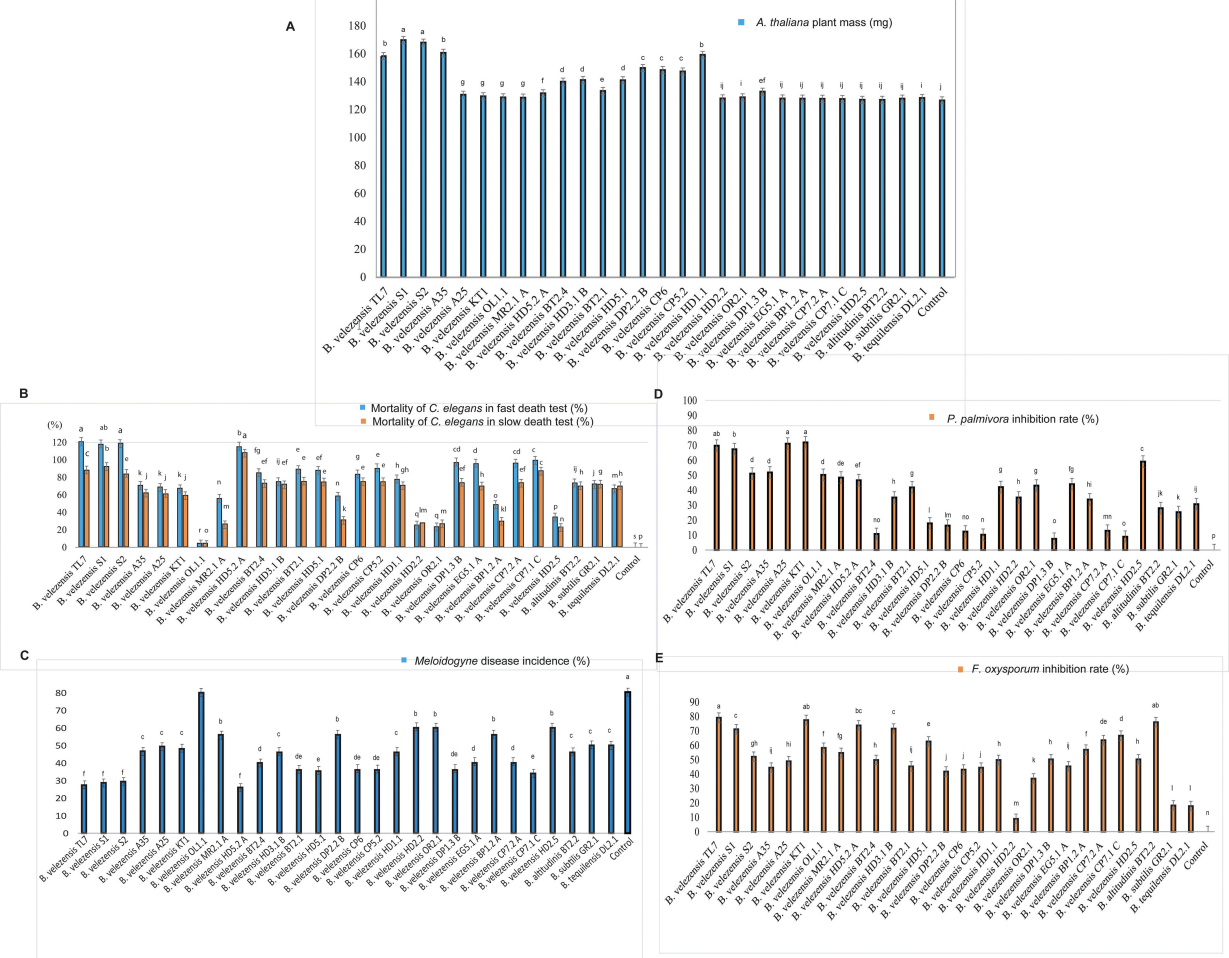
Strain evaluation for growth promotion, antagonistic and nematicidal activity. **(A)**: *Arabidopsis thaliana* growth promotion assay (Budiharjo et al., 2014). The columns in the diagram represent the fresh weight obtained after 21 days growth under controlled conditions in the growth chamber. **(B)**: Bioassay with *Caenorhabditis elegans*. Slow killing activity (slow death test) was determined on NGM plates, and fast killing activity (fast death test) in liquid medium. **(C)**: Determination of the biocontrol action of the *Bacillus* isolates on the root-knot nematode *Meloidogyne* sp. in greenhouse experiments. Tomato plants infested with *Meloidogyne* sp. were used for the test (counting of “knots” in tomato roots). The increase compared to the control without adding with the Bacillus isolate is shown. **(D)**: *In vitro* assay of antagonistic activity against *Phytophthora palmivora*. **(E)**: *In vitro* assay of antagonistic activity against *Fusarium oxysporum*. All diagrams showed the means of at least three replicates (n≥ 3). Negative controls were performed without treatment with the bacteria. Columns with superscripts with the same letter are not significantly different according to Fisher´s Least Significance Difference (LSD) Test (*p* ≤ 0.05). The LSD values were indicated as bars above the columns.

28 isolates, belonging to the *B. subtilis* species complex, were tested for their ability to promote plant growth in pot experiments performed with the model plant *Arabidopsis thaliana*. The *B. velezensis* isolates S1, S2, TL7, A35, and HD1.1 were found to stimulate plant growth based on biomass production by more than 25%. By contrast, the inoculation with the representatives of other species, such as *B. altitudinis* BT2.2, *B. subtilis* GR2.1, and *B. tequilensis* DL2.1 had no significant effect on growth of the *Arabidopsis* plantlets (**Figure 6A**; **Supplementary Table S9**).

Root-knot-forming nematodes of the genus *Melodogyne* are the main causative agents for the YLLR disease of coffee, and fast death disease of black pepper plants (Nguyen et al., 2016). A first screening for nematicidal activity was performed with the model nematode *Caenorhabditis elegans*, applying both the slow, and the fast killing-assay (see Materials and Methods)*. B. velezensis* isolates TL7, S1, S2, and HD5.2A were found most efficient in killing *C. elegans* under *in vitro* conditions (**Figure 6B**; **Supplementary Table S10**).

In planta experiments performed with tomato plants infested with second stage juveniles of *Meloidogyne* sp., isolated from roots of pepper plants, revealed that treatment with the isolates *B. velezensis* TL7, S1, S2, and HD5.2A were most efficient. They decreased the number of root-knots in the tomato plants infested with *Meloidogyne* sp. by around 65% (**Figure 6C**; **Supplementary Table S11**).

All isolates suppressed different plant pathogens known as the causative agents of important diseases in Vietnamese coffee and black pepper cultures, such as *Fusarium oxysporum*, and *Phytophthora palmivora*. *In vitro*-assays performed with all isolates revealed that the members of the *B. velezensis* species developed a strong antagonistic activity against the fungal pathogen *F. oxysporum*, and the oomycete *P. palmivora* Thereby, the *B. velezensis* isolates TL7, S1, and KT1 were found most efficient (**Figures 6D, E**; **Supplementary Table S10**).

**Figure 7:**
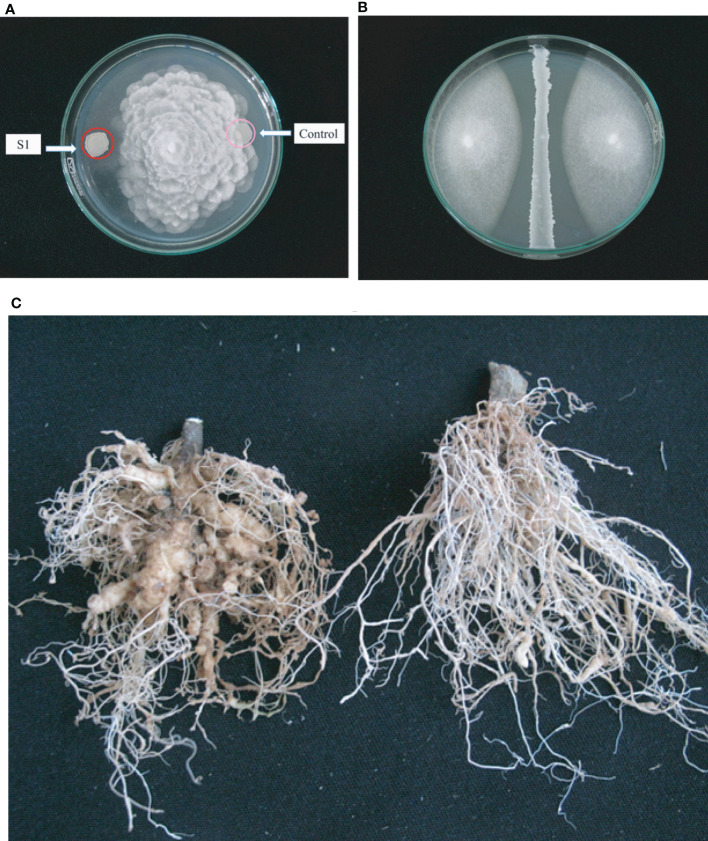
Antagonistic activity of *B*. *velezensis* S1 against fungal pathogens and nematodes. **(A)**: Qualitative assay of antagonistic activity of S1 (left side) exerted against the oomycete *P. palmivora* is indicated by growth inhibition of *P. palmivora* next to the bacterium. **(B)**: Semiquantitative assay of the inhibiting activity of S1 against *Fusarium oxysporum*. The diameter of the two fungal colonies growing to the left and to the right of the bacterial central line was found reduced compared to the control growing without bacteria. **(C)**: Demonstration of nematicidal activity. Formation of knots caused by *Meloidyne* sp. in tomato roots (left) is suppressed after treatment with *B*. *velezensis* S1 (right).

In summary, the *B. velezensis* isolates TL7 and S1 were found very efficient in promoting growth of the model plant *A. thaliana*, and in suppressing the main causative agents of Vietnamese crop plant diseases *F. oxysporum* (**Figure 7A**), *P. palmivora* (**Figure 7B**), and *Meloidogyne* sp. (**Figure 7C**). Both strains were selected for the further investigations performed in greenhouse and field trials.

### 3.4 Greenhouse and field trials performed with the *Bacillus velezensis* isolates TL7 and S1 corroborated their efficiency in stimulating growth and harvest yield under pathogen pressure

Due to their high biocontrol and plant growth-promoting activity, bioformulations of the endophytic *B. velezensis* TL7, and the soil borne *B. velezensis* S1 were chosen for the treatment of the coffee and black pepper plants in greenhouse and large field trials.

#### 3.4.1 Treatment with *Bacillus velezensis* S1 and TL7 reduced the pathogen pressure in coffee and black pepper plants

**Figure 8:**
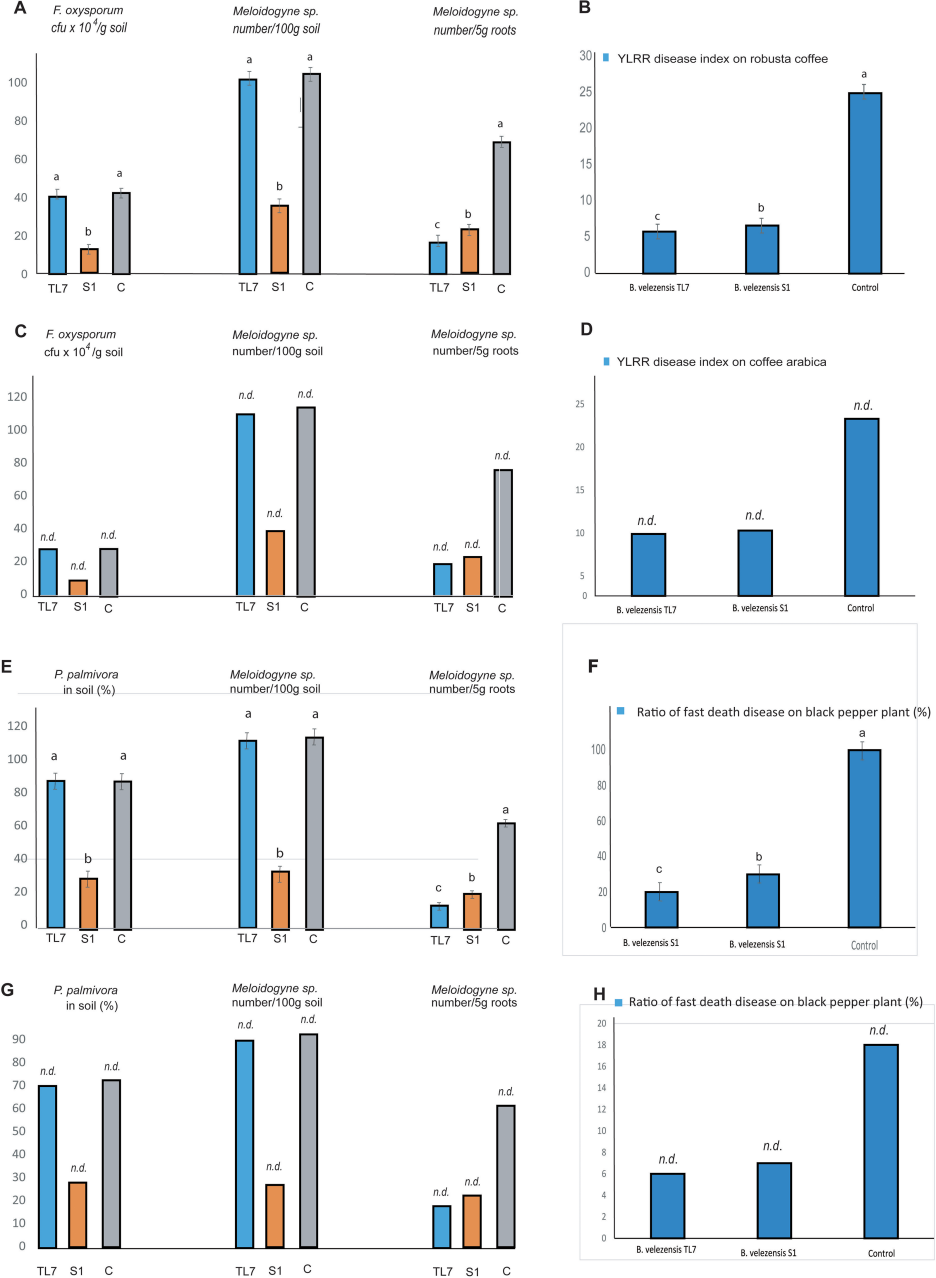
Effect of the treatment with *B*. *velezensis* TL7 and *B*. *velezensis* S1 on pathogens in coffee trees **(A–D)** and black pepper plants **(E–H)**. Samples were taken from the soil in vicinity of plant roots (“rhizospheric soil”) and the roots directly. The presence of the oomycete *Phytophthora palmivora* in soil was estimated indirectly by using the rose flower decolorizing test as described in Materials and Methods. **(A)**: Effect on the number of *F*. *oxysporum* (cfu x 10^4^/g soil) present in the rhizosphere and of *Meloidogyne* sp. (nematodes/5g root) present in the rizosphere and the roots of Robusta coffee trees (Vinh Linh variety) grown in greenhouse. The rate of reduction in comparison to the control without treatment is also presented. **(B)**: The YLRR disease index of Robusta coffee trees (Vinh Linh variety) grown in greenhouse, and treated with *B*. *velezensis* formulations was determined as described by Nguyen et al. (2016). **(C)**: Effect of inoculation with *B*. *velezensis* TL7 and S1 on the number of *F*. *oxysporum* (cfu x 10^4^/g soil) and *Meloidogyne* sp. (nematodes/100g soil and 5g root, respectively) infesting Arabica coffee trees grown in one-ha plots located in the Cau Dat, Xuan Truong, Da Lat, Lam Dong-mountain region. Five randomly selected areas containing six coffee trees each (a total of 30 plants) were used for analysis of the three variants (TL7, S1, and control). **(D)**: The YLRR disease index of Arabica coffee trees grown in one-ha plots located in the Cau Dat, Xuan Truong, Da Lat, Lam Dong mountain-region, and treated with the *B*. *velezensis* formulations. Five randomly selected areas containing a total of 100 plants were used for analysis of the three variants (TL7, S1, and control). **(E)**: Effect on the occurrence of *P. palmivora*, and the number of *Meloidogyne* sp. (nematodes/100g soil and 5g root, respectively) in black pepper plants grown in the greenhouse. The rate of pathogen reduction in comparison to the control without treatment is also shown. **(F)**: The incidence of fast death disease in black pepper plants grown in green-house was calculated as the quotient of the number of plants with symptoms of the fast death disease and the total number of black pepper plants multiplied with 100. A strong decrease of infested plants after treatment with *B*. *velezensis* TL7 and *B*. *velezensis* S1 was registered. **(G)**: Effect on the occurrence of *P. palmivora* and the number of *Meloidogyne* sp. in black pepper plants grown in 1 ha field plots in Chu Se, Gia Lai, Viet Nam. **(H)**: The incidence of fast death disease in black pepper plants grown in 1 ha field plots in Chu Se, Gia Lai, Viet Nam was drastically reduced after treatment with *B*. *velezensis* TL7 and *B. velezensis* S1. The mean values from the greenhouse experiments (n≥3) were depicted as columns. The error bars indicate the values calculated for the least significant difference (LSD). Bars with superscripts with the same letter are not significantly different according to Fisher´s LSD Test (*p* ≤ 0.05). The LSD values were indicated as bars at the top of the columns. Statistical analyses of the large-scale field trials (one ha plot per variant) were not performed (indicated on the bars by *n.d.*).

The effect of treatment with *B. velezensis* TL7 and *B. velezensis* S1 (7 x 10^10^ cfu/plant) on coffee trees infested with *Meloidogyne* sp., and *F. oxysporum* was investigated with Robusta coffee trees grown in greenhouse, and with Arabica coffee trees grown in one-ha mountain field plots. In the large field trials with 6,250 plants, 4 x 10^14^ cfu ha^-1^ of bioformulations were applied. The results obtained in greenhouse and field trials matched very well with each other: By treatment with the S1 bioformulation the number of *F. oxysporum* and of *Meloidogyne* sp. in the rhizosphere soil was reduced by more than 60%, whilst treatment with TL7 had no significant effect. However, the number of root-knots in coffee trees infested with *Meloidogyne* sp. was found to be drastically reduced after treatment with *B. velezensis* S1 and *B. velezensis* TL7, as well. Interestingly, the prevention rate of root-knots was found slightly higher, when the plants were treated with the endophytic TL7 before infested with the nematodes (**Figures 8A, C**; **Supplementary Tables S12**, **S14**).

For calculation of the disease index the plant phenotype (percentage of yellow leaves, occurrence of black rot on roots), and the number of root-knot-forming nematodes - but not the number of *F. oxysporum* - in the rhizospheric soil was used. According to this definition, the index reduction observed in plants treated with *B. velezensis* TL7 was similar as in plants treated with *B. velezensis* S1, despite that the treatment with *B. velezensis* S1 had a much higher impact on the presence of *F. oxysporum* in soil than the treatment with *B. velezensis* TL7 (**Figures 8B, D**; **Supplementary Tables S13**, **S15**).

The effect of the treatment with the bioformulations obtained from TL7 and S1 on the black pepper plants (Vinh Linh variety) infested with *P. palmivora*, and *Meloidogyne* sp. was investigated in greenhouse and large field trials as well. Also here, the results obtained in greenhouse and field trials matched very well.

Treatment of pepper plants with *B. velezensis* S1 formulation resulted in a strong decrease (around 70%) of *P. palmivora* in the soil samples obtained in vicinity of the black pepper plant roots. The occurrence of *Meloidogyne* sp. in roots was found to be reduced in the same range (60% – 70%) after treatment with the *B. velezensis* formulations (**Figures 8E, G**; **Supplementary Tables S16**, **S18**). The incidence of black pepper plants infested with the fast disease was lowered by 60-80% when treated with the *B. velezensis* bioformulations (**Figures 8F, H**; **Supplementary Tables S17**, **S19**).

#### 3.4.2 Treatment with *Bacillus velezensis* S1 and TL7 enhanced growth and harvest yield in coffee and black pepper plants

**Figure 9:**
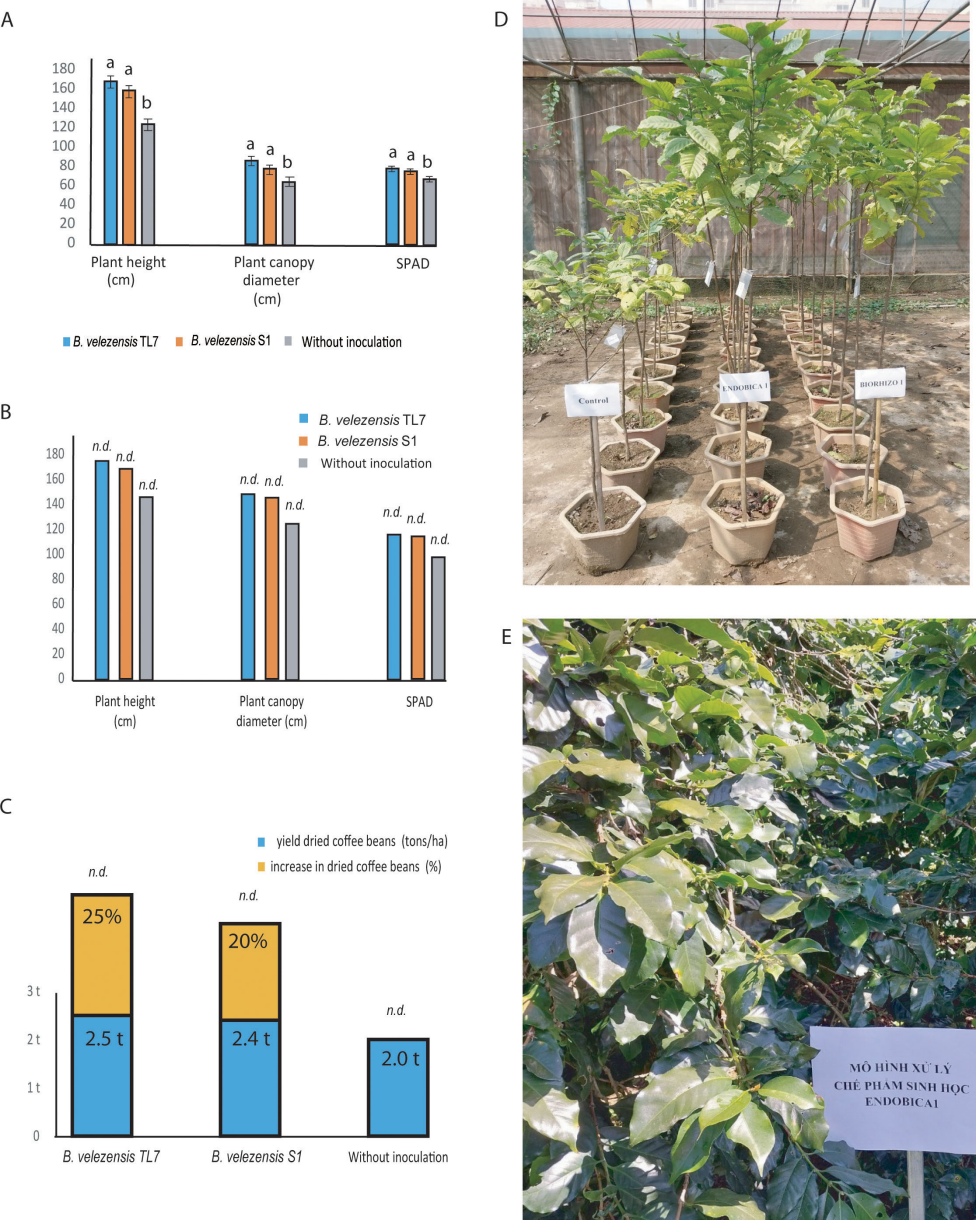
Growth promotion and enhancement of harvest yield of coffee trees by treatment with *B*. *velezensis* TL7 and *B*. *velezensis* S1 in greenhouse and large-scale field trials. **(A)**: Significant growth promotion of Robusta coffee trees inoculated with *B*. *velezensis* TL7, and *B*. *velezensis* S1grown in pots in the greenhouse. **(B)**: Growth stimulation of Arabica coffee trees grown in large field trials after inoculation with *B*. *velezensis* TL7, and *B*. *velezensis* S1 bioformulations. **(C)**: Increase of harvest yield in Arabica coffee trees grown under natural field conditions after treatment with *B*. *velezensis* TL7, and *B*. *velezensis* S1 bioformulations. **(D)**: Effect of treatment with *B*. *velezensis* TL7 (ENDOBICA1) and *B*. *velezensis* S1 (RHIZOBIO1) on Robusta coffee trees grown in greenhouse. **(E)**: Arabica coffee plantation plot (one ha) in Xuan Truong, Cau Dat, Da Lat, Lam Dong, Viet Nam (11°50’11.0”N 108°32’29.9”E). The plants were treated with a bioformulation prepared from *B*. *velezensis* TL7 (ENDOBICA1). Statistical analyses of the greenhouse experiments were performed as described in **Figure 8**. No statistical analysis was performed in large field trials (**B, C**). The bars without statistical analysis were labelled by *n.d*.

The greenhouse experiments performed with Robusta coffee trees demonstrated a significant increase of plant growth parameters, such as height, diameter, and chlorophyll content of leaves (SPAD), when the coffee trees were treated with the bioformulations (**Figures 9A, D**). The plant height was found enhanced by 34% after treatment with TL7, and by 27% after treatment with S1. The plant canopy diameter was enlarged by 32% (TL7), or 20% (S1). After treatment with the bacterial bioformulations, the SPAD values were increased by around 15% (TL7), or 11% (S1), compared to the control without treatment (**Supplementary Table S20**).

Large field trials performed with Arabica coffee trees in the Central Viet Nam-mountain region (altitude 1,500 – 1,600 m above sea level) corroborated the results obtained in the greenhouse experiments. Treatment with the bioformulations manufactured from *B. velezensis* TL7, and *B. velezensis* S1 resulted in an increase of the plant size (height and diameter), and of the chlorophyll content of the leaves in the same range as obtained in the greenhouse experiments (**Figures 9B, E**; **Supplementary Table S21**). In addition to the growth promoting effect observed for coffee trees treated with the *B. velezensis* bioformulations, a strong increase in the harvest yield of the Arabica coffee trees treated with *B. velezensis* TL7 or S1 was registered. The harvest yield of Arabica coffee beans exceeds the yield of the control without treatment, and the average yield obtained for this coffee variety in this region by 20 – 25% (**Figure 9C**; **Supplementary Table S22**).

**Figure 10:**
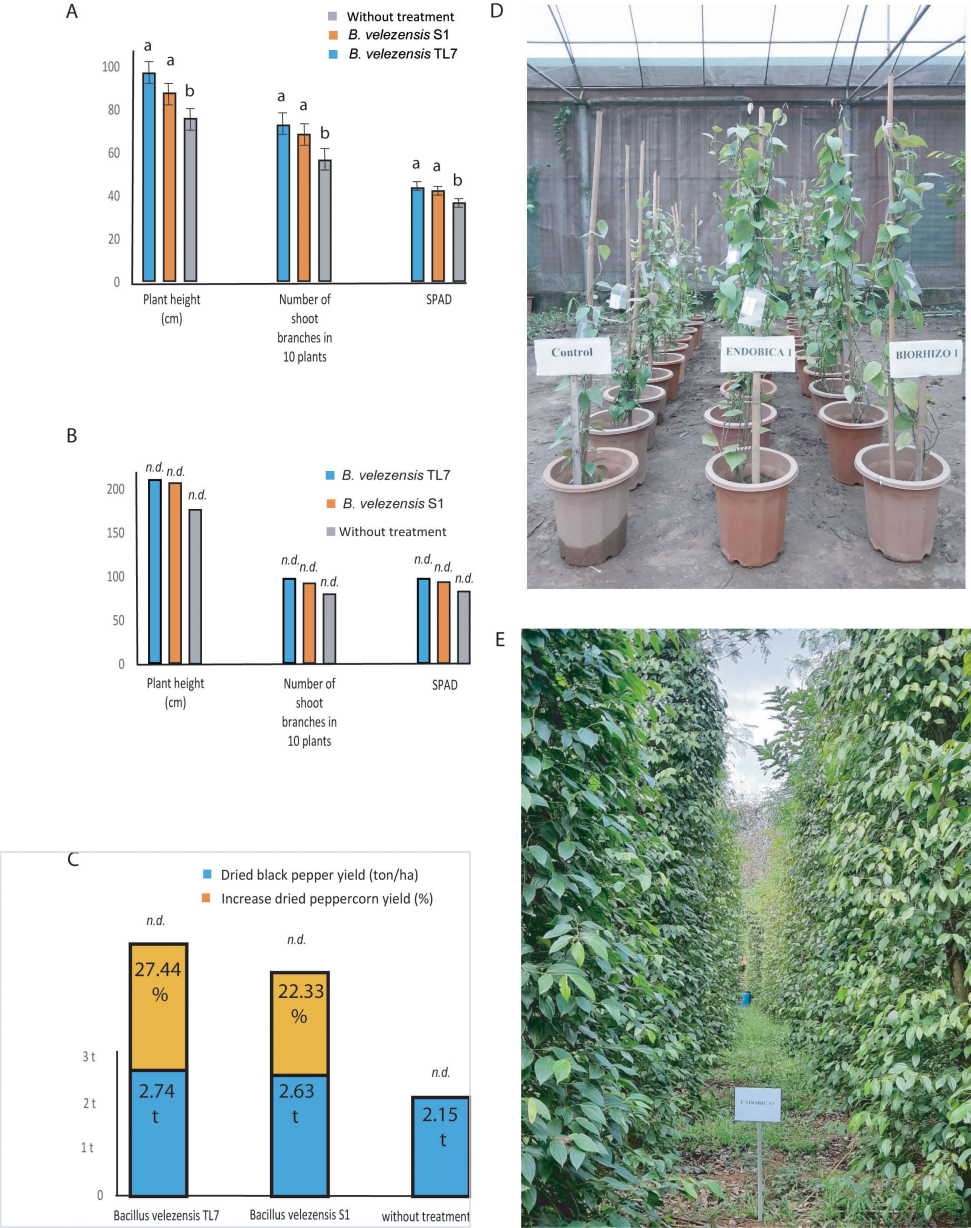
Growth promotion and enhancement of harvest yield of black pepper plants treated by *B*. *velezensis* TL7 and *B*. *velezensis* S1 in greenhouse and large-scale field trials. **(A)**: Significant growth promotion of the plants inoculated with *B*. *velezensis* TL7, and *B*. *velezensis* S1 grown in pots in the greenhouse. **(B)**: Growth stimulation of plants grown in large field trials (Chu Se, Gia Lai) after inoculation with *B*. *velezensis* TL7, and *B*. *velezensis* S1 bioformulations. **(C)**: Increase of harvest yield in black pepper plants grown under natural field conditions (one-ha plots, Chu Se, Gia Lai) after treatment with *B*. *velezensis* TL7, and *B*. *velezensis* S1 bioformulations. **(D)**: Effect of treatment with *B*. *velezensis* TL7 (ENDOBICA1) and *B*. *velezensis* S1 (RHIZOBIO1) on pepper plants grown in greenhouse. **(E)**: Black pepper plantation plot (1 ha), Chu Se, Gia Lai, Viet Nam (13°39’27.6”N 108°06’42.6”E). The plants were treated with a bioformulation prepared from *B*. *velezensis* TL7 (ENDOBICA1). Statistical analyses of the greenhouse experiments were performed as described in **Figure 8**. No statistical analysis was performed in large field trials (10B, 10C). The bars without statistical analysis were labelled by *n.d*.

Likewise, as in coffee trees, the effect of the bioformulations on black pepper plants (variety Vinh Linh) was tested in greenhouse and field trials. Plant growth parameters, such as plant height, number of side-branches, and the chlorophyll content in leaves (SPAD) was found to be enhanced in greenhouse plants treated with the bacteria formulations (**Figures 10A, D**; **Supplementary Table S23**). The large field trials (one-ha plots per experimental variant) performed in the Chu Se, Gia Lai-mountain region (700 – 800 m above sea level) confirmed the results obtained in the greenhouse. All growth parameter were found enhanced in a similar range after treatment with *B. velezensis* TL7 and S1 (**Figures 10B, E**; **Supplementary Table S24**). Similar as in the coffee trees, the harvest yield on peppercorns was found to be enhanced after treatment with the bioformulations by more than 20% (**Figure 10C**; **Supplementary Table S25**).

## 4 Discussion

Due to the growing concern about utilization of chemically synthesized fertilizers and pesticides in agriculture, and the consumer demand for food security, their substitution by environmentally friendly biologicals, able to improve crop performance, is a pressing need (Stukenbrock and Gurr, 2023). However, partial or complete substitution of agrochemicals by biologicals, for example biocontrol agents and biostimulants, is a cost-intensive and challenging process. Many plant-beneficial microbes, which were found efficient under controlled laboratory and greenhouse conditions, failed or delivered inconsistent results under field conditions (Besset-Manzoni et al., 2019). We described here an approach for selecting suitable microbial candidates for developing efficient bioagents preferentially utilized in Vietnamese coffee, and black pepper production as integral part of the concept of Good Agriculture Practice (Dao et al., 2015). Our selection procedure based on the following steps (i) isolation of endospore forming bacteria from healthy plants grown in pathogen infested crop fields; (ii) *in vitro* test for their efficacy against the main local pathogens negatively affecting coffee and black pepper growth (nematodes, fungi, and oomycetes); (iii) genome analysis from 59 isolates for taxonomical assignment and predicting their potential for synthesizing secondary metabolites efficient against plant pathogens; (iv) in planta assays for determining the potential of 30 representatives of the *B. subtilis* species complex to suppress plant pathogens and to promote plant growth under pathogen pressure. Based on this strategy, two plant associated isolates, TL7 and S1, highly similar in their genome sequence with the biocontrol model strain *B. velezensis* FZB42, were selected and successfully applied in large field trials.

In our previous study (Tam et al., 2020) endospore-forming representatives of different genera and species were isolated from Vietnamese crop plants. Representatives of *Brevibacillus* spp. have a rich arsenal of bioactive peptides. They were found efficient in plant growth promotion and biocontrol in laboratory and greenhouse experiments (Jähne et al., 2023). However, due to their relatively slow growth rate under large scale conditions, manufacturing of bioformulations from Brevibacilli appears not economically appropriate. By contrast, many plant-associated representatives of the *B. subtilis* species complex (Fritze, 2004) were found suitable for large scale production of durable endospores and manufacturing bioagents under economically feasible conditions (Borriss, 2011). In this study we could show, that several *B. velezensis* isolates from Vietnamese crop plants were promising candidates for large-scale application in sustainable agriculture.

In recent years, it became increasingly evident that *B. velezensis* is by far the most important species for developing commercial biocontrol and growth-stimulating agents (Vallejo, 2023). However, for several reasons, this understanding has not been generally recognized. The studies performed by Borriss et al. (2011), and Dunlap (2019) demonstrated that most strains used for commercial agents were registered under inconsistent species names. For example, nine *B. velezensis* strains were registered as *B. subtilis* or *B. amyloliquefaciens*. Misclassification of commercial strains is mainly due to the insufficient species resolution of the members of the *B. subtilis* species complex, when solely based on phenotypic characteristics, and the highly conserved 16S rRNA sequence (Rooney et al., 2009). Despite that the majority of these strains have now been genome-sequenced, and their taxonomical boundaries can be corrected, the companies prefer to keep their “old” species names in order to avoid additional registration efforts and possible confusion of their customers. For example, *B. subtilis* GB03, registered as the biopesticide “Kodiak” by the EPA in 1992, is still referred as *B. subtilis*, despite its close relationship with *B. velezensis* FZB42 was clearly demonstrated (Choi et al., 2014). Comparative field trials performed with 13 strains representing different species of the *B. subtilis* species complex revealed that *B. velezensis* FZB42 exhibits the highest effect on growth and harvest yield of maize and potato plants, thereby surpassing *B. subtilis*, *B. atrophaeus*, and other species (Mülner et al., 2020).

*B. velezensis*, formerly designated as *B. amyloliquefaciens* subsp. *plantarum* (Borriss et al., 2011), forms together with *B. amyloliquefaciens* (Priest et al., 1987) and *B. siamensis* (Sumpavapol et al., 2010) the “operational group *B. amyloliquefaciens*”, whose genome similarities are slightly below the ANI cut off of 95-96% for species delineation (Fan et al., 2017). The three species are distinguished by their life style, and by the number of gene clusters involved in biosynthesis of important secondary metabolites (BGCs, Kautsar et al., 2020). The plant-associated *B. velezensis* FZB42 devotes nearly 10% of its genomic capacity to the synthesis of antimicrobial peptides, and polyketides including bacillaene, macrolactin, and difficidin. It is able to promote plant growth, and to suppress plant pathogens (Chen et al., 2007). By contrast, the closely related soil bacterium *B. amyloliquefaciens* is unable to synthesize the polyketides macrolactin, and difficidin, and does not possess numerous hydrolases, present in *B. velezensis*, which are involved in degradation of plant cell material, such as the ß-1,4 endo-glucanases EglS, and BglC, or the xylanases XylA, and XynA (Rückert et al., 2011, **Supplementary Table S26**). *B. siamensis* strains were isolated mainly from plant food products. They do not harbor the giant gene cluster for macrolactin synthesis, but are able to synthesize the polyketides bacillaene and difficidin (Fan et al., 2017). During our screening procedure for plant-associated endospore-forming bacteria with potential to suppress plant pathogens, we could only detect representatives of *B. velezensis*, but not the other representatives of the *B. amyloliquefaciens* operational group, suggesting that the latter species have no important role in the plant microbiome. For this reason, we focused our subsequent work mainly on the representatives of the *B. velezensis* isolates obtained from inside of different plant organs (endophytes), and from the plant rhizosphere (rhizobacteria). However, unique genes connected with the plant-associated life-style were not detected, when the singletons in the genomes of the 27 *B. velezensis* isolates were analyzed. Also functional category analysis revealed no apparent differences between the rhizosphere inhabitants, and the endophytes. We assume, that the ability of endophytes to cross plant barriers might be rather due to the expression level of genes enabling the bacteria to overcome plant stress responses, than to the presence of specific genes only occurring in endophytes.

Genome analysis revealed a striking similarity of the majority of the *B. velezensis* isolates with *B. velezensis* FZB42 (**Figure 1**), isolated from healthy plants growing within a pathogen-infested German sugar beet field (Krebs et al., 1998), suggesting that bacterial strains harboring closely related genomes can be isolated from very remote geographical regions. A core of 15 biosynthetic gene clusters (BGCs), encompassing clusters for non-ribosomal synthesis of lipopeptides (surfactin, fengycin, iturins), other peptides (bacillibactin, bacilysin), polyketides (macrolactin, bacillaene, difficidin, two unknown T1- and T3-polyketides), ribosomal synthesized peptides such as the LCI antimicrobial peptide, and amylocyclicin, two different terpenes, and the synthesis genes for four different types of the competence pheromone ComX, were detected (**Figure 4**). In total, 36 BGCs were detected in the genomes of the 30 representatives of the *B. subtilis* species complex investigated in this study (**Supplementary Table S8**).

Non-ribosomal synthesis of cyclic lipopeptides with antifungal action, such as iturins, and fengycins is common in different representatives the *B. subtilis* species complex (Dunlap et al., 2019). The *B. velezensis* isolates investigated in this study harbored BGCs for synthesis of different iturinic lipopeptides, such as bacillomycin D, iturin A, and bacillomycin L. *B. tequilensis* DL2.1 harbored a gene cluster predicted to encode the iturinic heptapeptide mojavensin. Together with fengycin, these compounds were detected directly in the isolates applying MALDI-TOF MS. We assume, that the antagonistic effect of the *B. velezensis* isolates against the fungal pathogen *F. oxysporum* was mainly due to the production of cyclic lipopeptides, especially bacillomycin D or other iturinic lipopeptides, whilst the suppression of *P. palmivora* might be due to bacilysin. There are many reports about the antagonistic action of cyclic lipopeptides against fungal pathogens including *F. oxysporum* (Koumoutsi et al., 2004; Chowdhury et al., 2015), one of the causative agents of the coffee YLRR disease. Non-ribosomal-synthesized polyketides (Chen et al., 2006), and the dipeptide bacilysin are known for their antibacterial action against the causative agent of fire blight disease on orchard trees, *Erwinia amylovora* (Chen et al., 2009a), and the rice pathogen, *Xanthomonas oryzae* (Wu et al., 2015). Recently, it was demonstrated that bacilysin from FZB42 antagonizes *Phytophthora sojae* and other representatives of the *Phytophthora* genus including *P. palmivora* (Han et al., 2021), one of the causative agents of black pepper diseases.

Besides their direct antagonistic effect on growth of plant pathogens, secondary non-ribosomally synthesized peptides can trigger the plant defense response known as induced systemic resistance (ISR). It has been early shown that purified surfactin, and to a minor extent fengycin, elicited the ISR-dependent plant immune resistance against fungal pathogens (Ongena et al., 2007). Mutants of FZB42, only able to produce surfactin, but no other antimicrobial peptides, were shown to induce systemic resistance against *Rhizoctonia solani* in lettuce plants (Chowdhury et al., 2015). Surfactin of *B. velezensis* was shown to be essential for colonization, biofilm formation on tomato root and leaf surfaces and subsequent protection (ISR) against *Botrytis cinerea* (Stoll et al., 2021).

Two gene clusters predicted to be involved in ribosomal synthesis of RiPPs were found conserved in all *B. velezensis* isolates. The head-to-tail-cyclized amylocyclicin, first described to occur in FZB42, was reported to display a high antagonistic activity against some related Gram-positive bacteria (Scholz et al., 2014). The antimicrobial peptide LCI have strong antibacterial activity against *Xanthomonas campestris*, and *Pseudomonas solanacearum* (Gong et al., 2011). One of the two terpene gene clusters detected in the *B. velezensis* isolates was encoding sporulene, discovered by Kontnik et al. (2008) in *B. subtilis.* The sporulene heptaprenyl lipids, synthesized by squalene-hopene cyclase, contributes to the resistance of spores to reactive oxygen species (Bosak et al., 2008). As in *B. subtilis*, the ability to uptake DNA (genetic competence) is controlled by an isoprenylated small peptide with variable amino acid sequence, the ComX pheromone. The signaling molecule ComX is synthesized as an inactive precursor, and is then cleaved and modified by ComQ before export to the extracellular environment, and its sensing by the ComP-ComA two-component system (Ansaldi et al., 2002). *B. velezensis* isolates possessed variable ComX precursor sequences. Four different variants were distinguished within the 27 isolates, which determine the specificity of the quorum-sensing system within the species (**Figure 4**; **Supplementary Figure S8**).

In addition to the 15 BGCs conserved in all *B. velezensis* isolates, eight were found sporadically distributed in some representatives of the species. The *nrs* gene cluster occurred in the genomes of most, but not all *B. velezensis* isolates. The product of the *nrs* gene cluster was unknown for long time, and has been recently identified as bacillothiazole. The compound is non-ribosomally synthesized, and modified by a discrete oxidase encoded by the *nrs* gene cluster (Shen et al., 2022). Most of the BGCs encoded ribosomally-synthesized and post-translationally modified peptides (RiPPs), such as plantazolicin (Scholz et al., 2011), and several classes of lanthipeptides. Plantazolicin was detected in the group of *B. velezensis* isolates, closely related to FZB42, including TL7 and S1 (**Supplementary Figure S7**). BGCs for synthesis and modification of class ii- and class iv-lanthipeptides were detected in a few *B. velezensis* isolates (**Supplementary Figure S12**), whilst the gene cluster for synthesis of a sactipeptide (sulfur-to-alpha carbon thioether cross-linked peptides) of unknown function was very common in most of the *B. velezensis* isolates, except BP1.2A, and BT2.4 (**Figure 4**). The gene cluster was similar to the uncharacterized sactipeptide gene cluster in *B. altitudinis* BT2.2, and the subtilosin A gene clusters detected in *B. subtilis* GR2.1 and *B. tequilensis* DL2.1 (**Supplementary Figure S11**). A gene cluster encoding a representative of the thiocillin family RiPP (WP_109955211.1) was detected in *B. velezensis* CP6 (**Supplementary Figure S13**). Thiocillins have been shown to act as signaling molecules stimulating biofilm formation in *B. subtilis* (Bleich et al., 2015). The *B. altitudinis* BT2.2 genome proved as a rich source of BGCs encoding known and unknown RiPPs, such as the head-to-tail cyclized peptides pumilarin, and the BhlA/UviB peptide, which is resembling the enterocin AS-48 (BGC0000489), and the class ii bacteriocin aureocin A53, which is resembling lacticinQ/lacticin Z (**Supplementary Figure S14**). Whilst the importance of the non-ribosomal synthesized peptides as direct antagonists of bacterial and fungal pathogens, and as elicitors of plant ISR is without doubt, the function of most RiPPS is still less understood, and seems to be more complex. Besides their possible role in direct competition with the other members of the local microbiome, they might be important for governing the cell behavior, such as biofilm formation, regulating of cell density and genetic competence, and manyfold interactions with the environment.

Besides their suppressing effect on microbial pathogens, significant antagonistic actions of the *B. velezensis* isolates against root-knot nematodes were registered in laboratory and field trial experiments. This is especially important, because the root-knot nematode *Meloidogyne incognita* was identified as being the main causative agent of the coffee and black pepper diseases in Vietnam (Trinh et al., 2022). Different metabolites produced by *B. velezensis* were reported to be responsible for the antagonistic action exerted by this species against nematodes. By screening a random mutant library of FZB42 generated by the mariner transposon TnYLB-1, plantazolicin was identified as being involved in the antinematode effect of this bacterium (Liu et al., 2013). In addition, antinematode compounds recently identified in *B. velezensis* were thymine and the volatile compound hexahydropyrrolo [1,2-a] pyrazine-1,4-dione. The compounds were predicted to interact with acetylcholinesterase (Trinh et al., 2022). Volatiles of *B. atrophaeus* GBSC56, another member of the *B. subtilis* species complex, were shown to stimulate ISR in tomato against *M. incognita* (Ayaz et al., 2021). Nematicidal volatiles of GBSC56, FZB42, and *B. subtilis* SYST2 caused high killing rates due to production of reactive oxygen species (ROS) in the plant parasitic nematode *Aphelenchoides besseyi* (Ali et al., 2023).

Besides their impressive function in biocontrol of plant pathogens, promotion of plant growth was also observed, when the plants were treated with *B. velezensis* TL7 and S1. After an extensive genome analysis, a careful checking of their ability to produce antimicrobial metabolites, and their ability to enhance plant growth, and to suppress the most important local plant pathogens in laboratory scale, we choosed *B. velezensis* strains with different life style, the rhizobacterium S1, and the endophyte TL7, for greenhouse experiments, and large-scale field trials. Both isolates resembled in their genome sequence very much each other and FZB42 (ANI: ≥ 99.96%), but can be distinguished by the presence or absence of the bacillothiazole and the sactipeptide gene cluster (**Figure 4**). Proving efficacy in large field trials is the number one issue in the development line for better microbial agents having at least the same performance under field conditions as the chemicals they have to replace (Waltz, 2023).

Our greenhouse and field trial results obtained for two important Vietnamese crops, black pepper and coffee trees, demonstrated that treatment with the two selected *B. velezensis* strains, despite of their different plant-associated life-style, had a strong impact on growth and harvest yield. The harvest yields determined under natural farming conditions were found to be increased by more than 20% compared to the untreated control (**Figures 9**, **10**). In a previous study an increase in harvest yield of 4.5% was obtained after a combined application of rhizosphere and endophytic bacteria in black pepper plants growing in selected farms in the Central Highlands of Vietnam (Nguyen et al., 2021). Simultaneously, we could show that growth promotion, and increase of harvest yield were closely connected with the ability of the *B. velezensis* strains TL7 and S1, to reduce the disease rate of the pathogen infested coffee trees, and the black pepper plants. Besides fungal and oomycetes pathogens, our main focus in the large field trials, and also in the greenhouse experiments, was directed on the occurrence of the root knot nematode *Meloidogyne* sp. It ruled out that treatment with the two *B. velezensis* strains resulted in different effects on the presence of the pathogens in the rhizosphere soil. Whilst the endophyte TL7 had virtually none effect on the number of nematodes, and the other pathogens present in the soil in vicinity of the plant roots, application of the rhizobacterium S1 reduced the number of pathogens by ≥ 60%. However, both, the TL7 endophyte, and the S1 rhizobacterium, were found very efficient in suppressing the root-knot nematodes inside of the roots, suggesting that both strains were similar efficient in decreasing the disease rates in black pepper, and coffee trees, as well. Bioformulations containing the endospores from selected *B. velezensis* isolates, applied within a holistic approach of Agriculture Good Manufacturing Practice, will contribute to further diminishing the use of harmful agrochemicals in coffee and black pepper plantations in Vietnam.

## 5 Conclusion

*B. velezensis* isolates from healthy Vietnamese crop plants grown in pathogen-infested environments have a high potential to enhance harvest yield of coffee trees, and black pepper plants, also under condition of high pathogen pressure mainly exerted by root-knot nematodes, plant parasitic fungi, and oomycetes. We could demonstrate that after a comprehensive genome analysis, and applying screening procedures for biocontrol and plant growth promotion, it was possible to select promising candidates for large-field trials. We could show that the bioformulations manufactured from durable endospores of the *B. velezensis* isolates TL7, and S1 were able to suppress plant pathogens, and to enhance growth and harvest yield of main Vietnamese crop plants under the conditions of large-field trials performed in their main cultivation regions.

## Data availability statement

The datasets presented in this study can be found in online repositories. The names of the repository/repositories and accession number(s) can be found in the article/**Supplementary Material**.

## Author contributions

LT: conceptualization, project administration, methodology, data curation, writing-original draft. JJ, PTL, LP, CB, AS, SH, JV, LN, PM: investigation, methodology, validation. JB: software. HT: methodology. MW and TH: formal analysis, validation, PM and TS: data curation. PH and NL: project administration. HT and LC: conceptualization, methodology. PL and TS: conceptualization, supervision, project administration. RB: conceptualization, project administration, writing-original draft, writing-review and editing. All authors contributed to the article and approved the submitted version.
