# Supplementary material for: Two plant-associated Bacillus velezensis strains selected after genome analysis, metabolite profiling, and with proved biocontrol potential, were enhancing harvest yield of coffee and black pepper in large field trials
Source: Front Plant Sci. 2023 Jun 23;14:1194887. doi: 10.3389/fpls.2023.1194887 (PMC10327441; doi:10.3389/fpls.2023.1194887)
Supplement: Supplementary file 1 [file DataSheet_1.pdf]

Bacillus velezensis strains isolated from Vietnamese crop plants are efficient in phytostimulation and biocontrol of plant pathogens

Le Thi Thanh Tam[1], Jennifer Jähne[2], Pham Thi Luong[1], Le Thi Phuong Thao[1], Le Mai Nhat[3], Christian Blumenscheit[2], Andy Schneider[2], Jochen Blom[4], Markus Weinmann[5], Le Thi Kim Chung[6], Pham Le Anh Minh[7], Ha Minh Thanh[1], Trinh Xuan Hoat[1], Pham Cong Hoat[8], Tran Cao Son[9], Joachim Vater[2], Nguyen Van Liem[1], Thomas Schweder[10,11], Peter Lasch[2], Rainer Borriss[10,12*]

Supplemental Figures

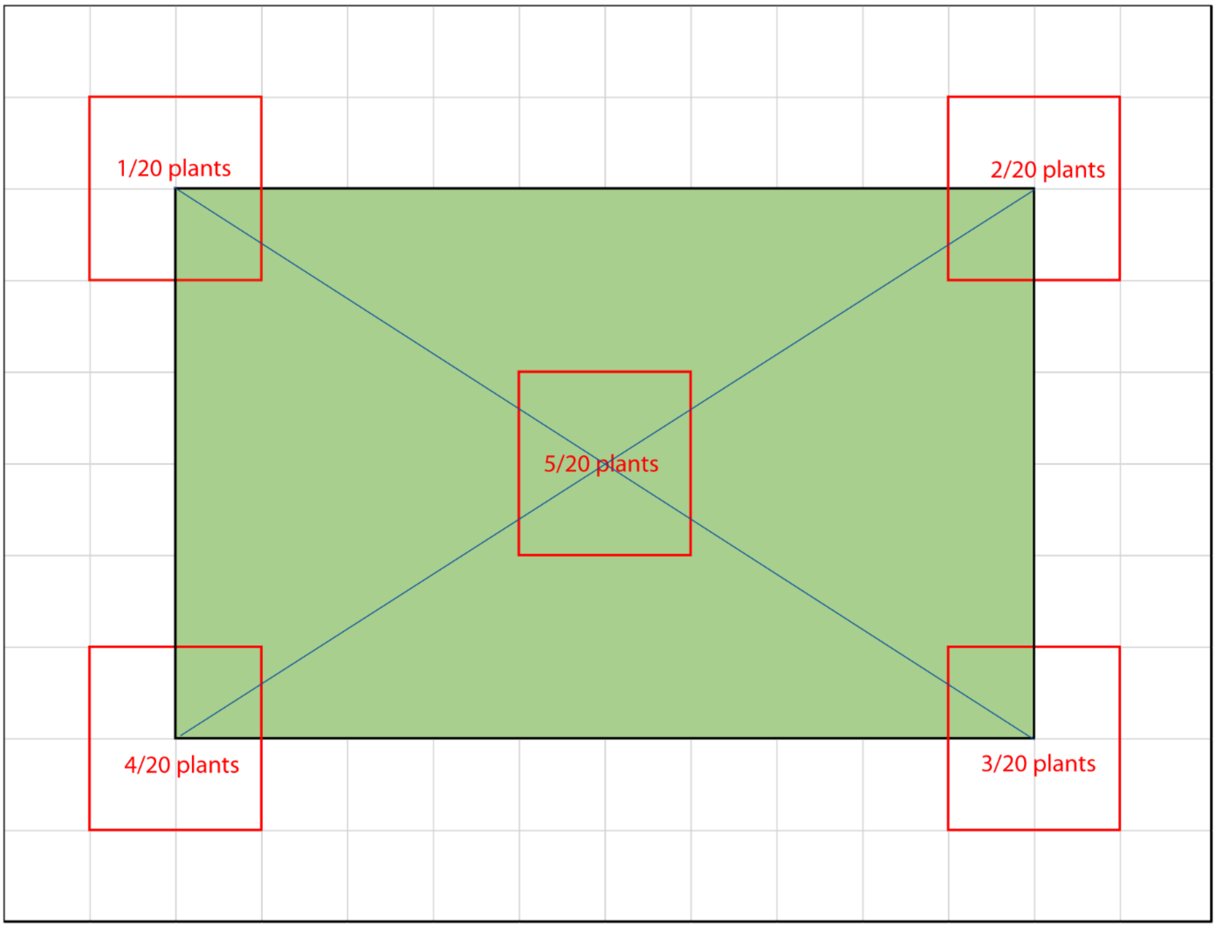

Suppl. Fig. S1: Field trials were performed in one-hectare plots/variant. Five selected areas with 20 plants each were used for data collection.

-

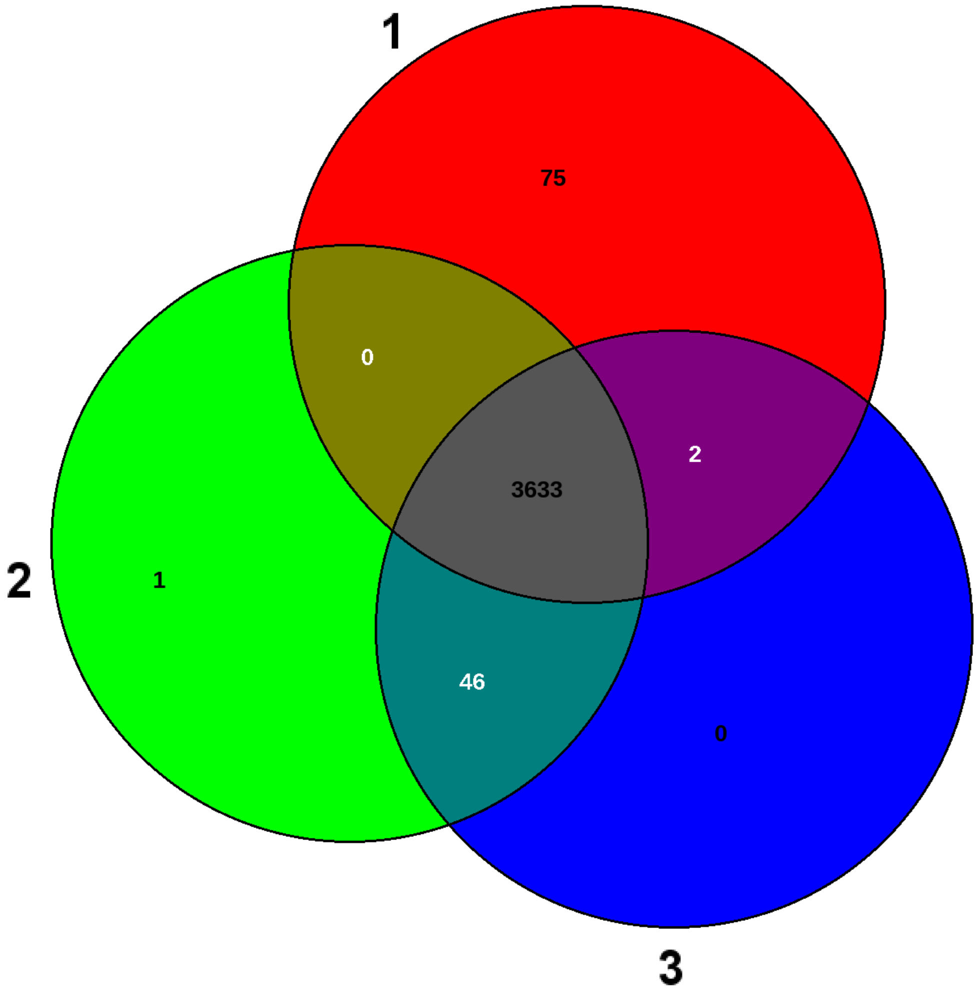

1: Bacillus_velezensis_FZB42_CP000560
2: Bacillus_velezensis_strain_BP1_2A_CP085504
3: Bacillus_velezensis_strain_BT2_4_CP085505

Suppl. Fig. S2: Venn diagram FFZB42 (1), BP1.2A (2), and BT2.4 (3).

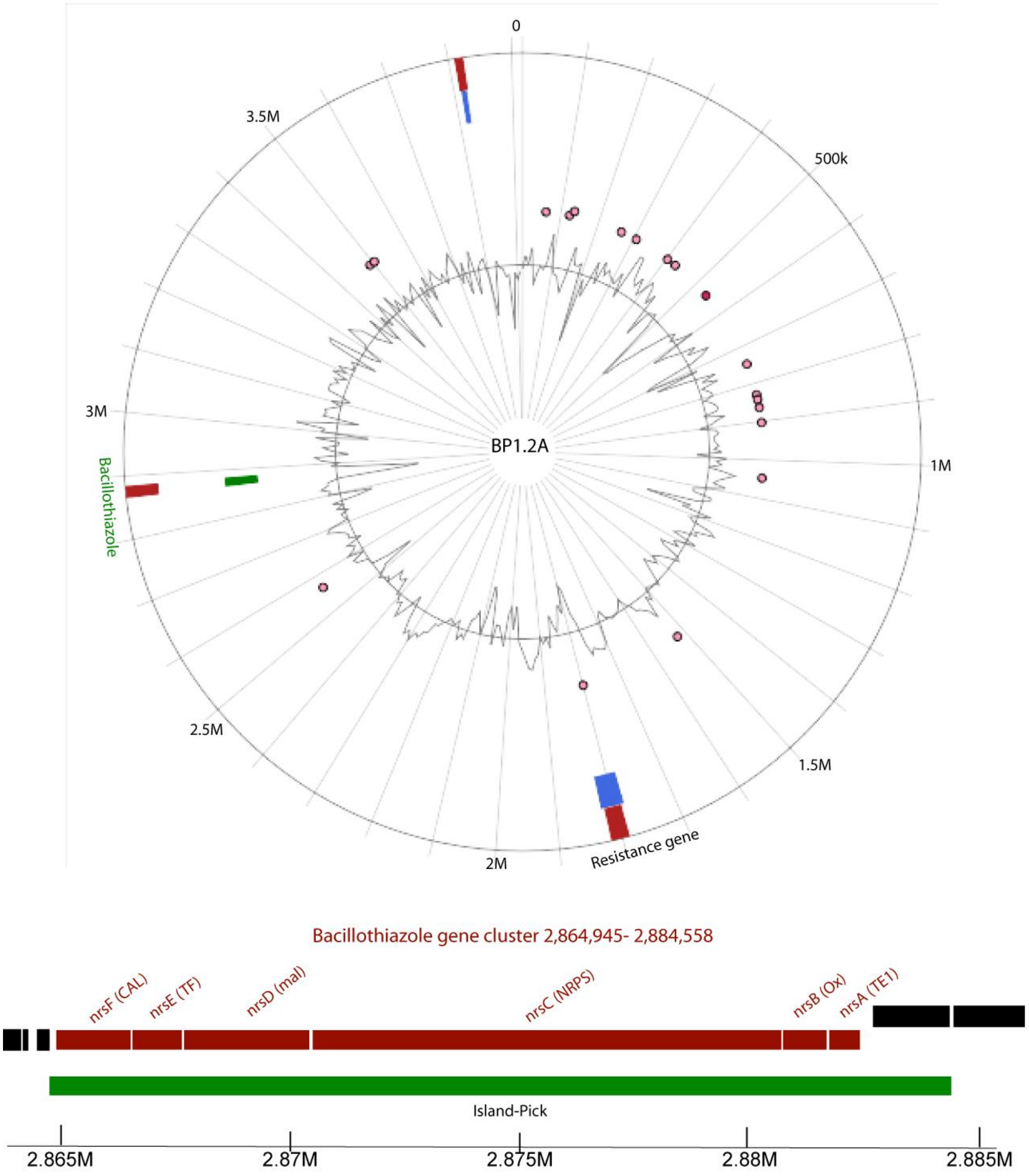

Suppl Fig. S3: The genomic island at 2.86-2.88M in *B. velezensis* BP1.2A predicted by Island-Pick harbored the complete bacillothiazole gene cluster.

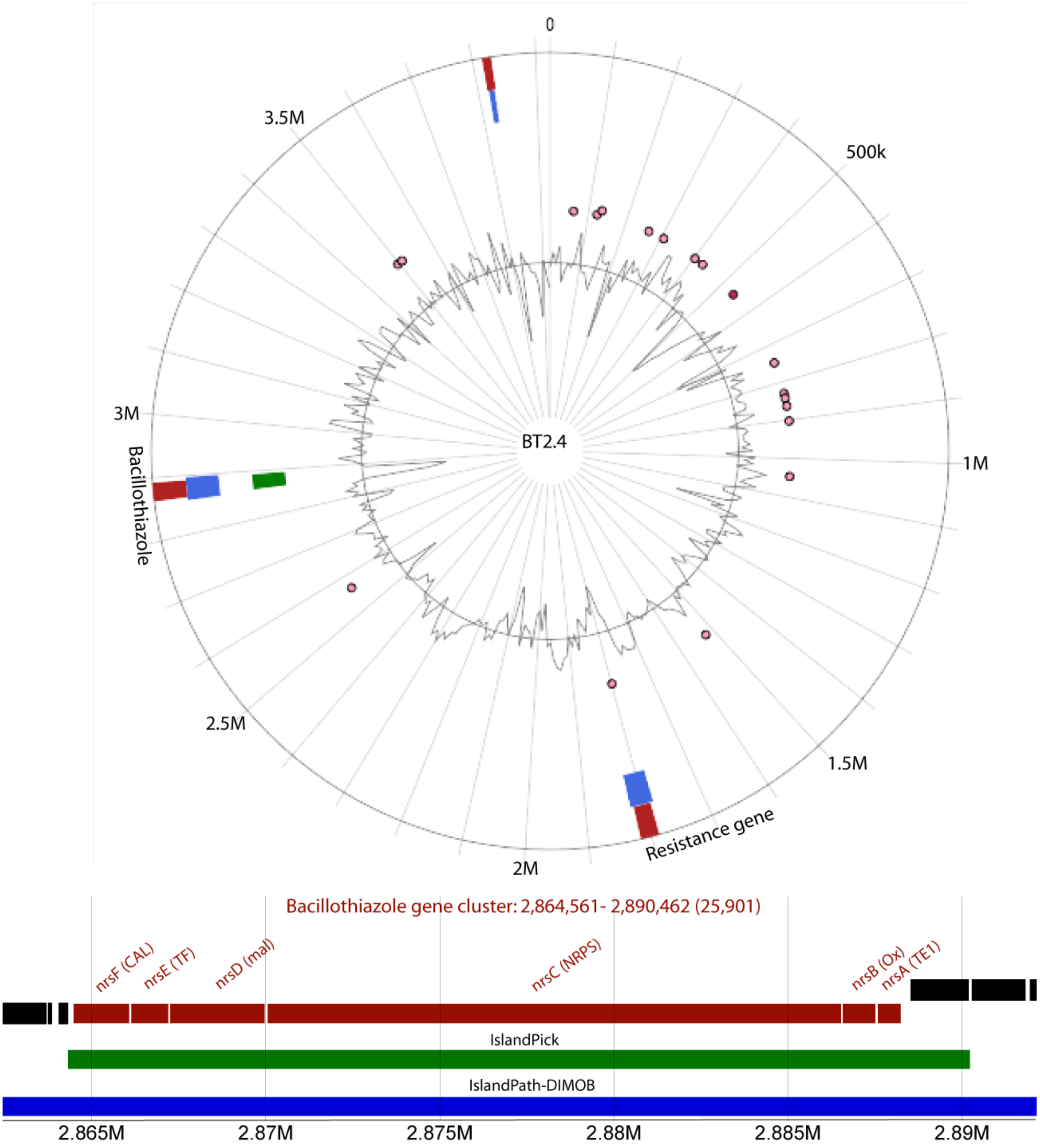

Suppl Fig. S4: The genomic island at 2.86-2.89M in *B. velezensis* BT2.4 predicted by Island-Pick and IslandPath-DIMOB harbored the complete bacillothiazole gene cluster.

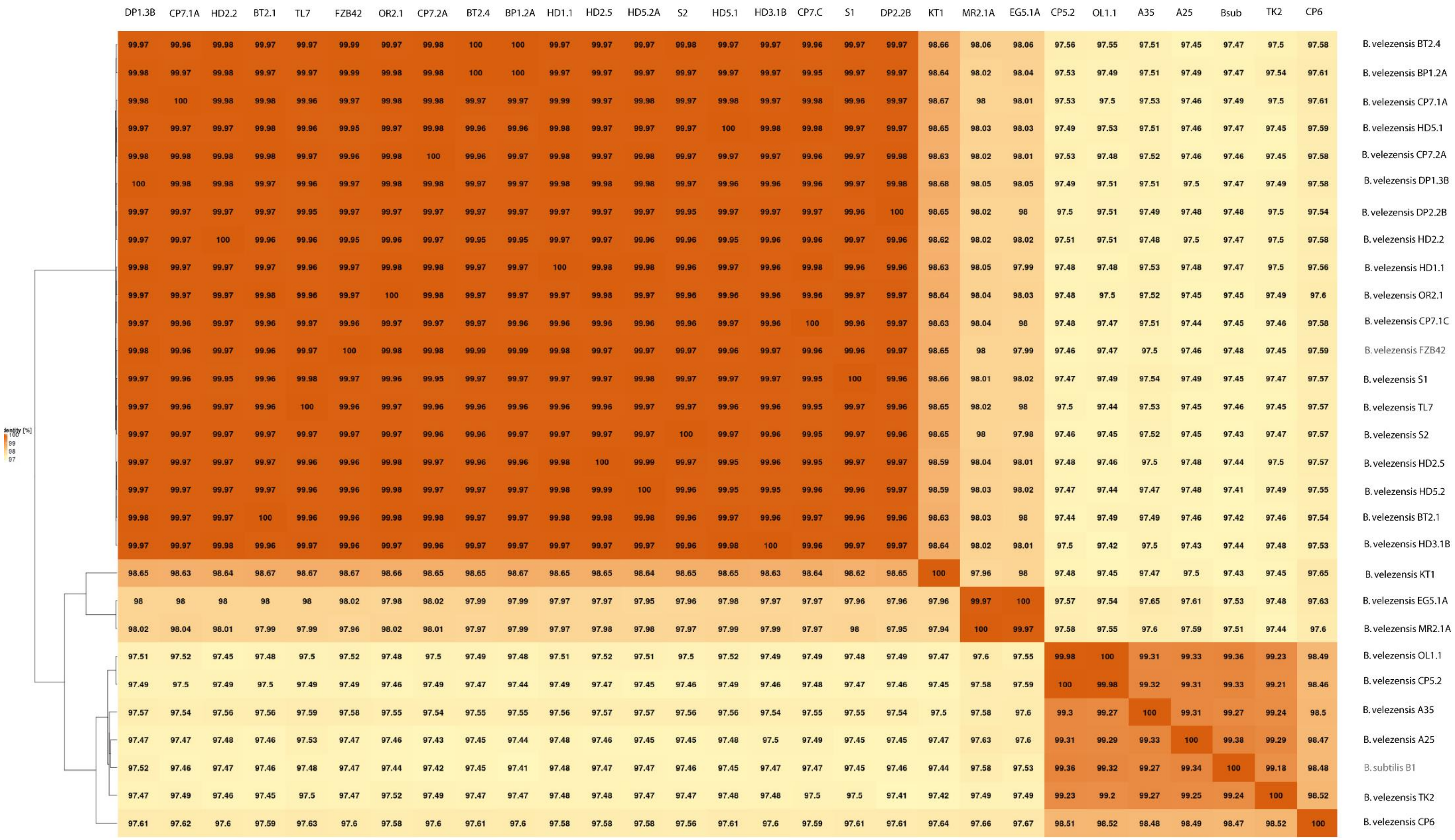

Suppl. Fig. S5: FastANI heatmap (https://pubmed.ncbi.nlm.nih.gov/30504855/) of the 27 *B. velezensis* isolates from Vietnamese crop plants. *B. velezensis* FZB42 and *B. subtilis B1* were included for comparison.

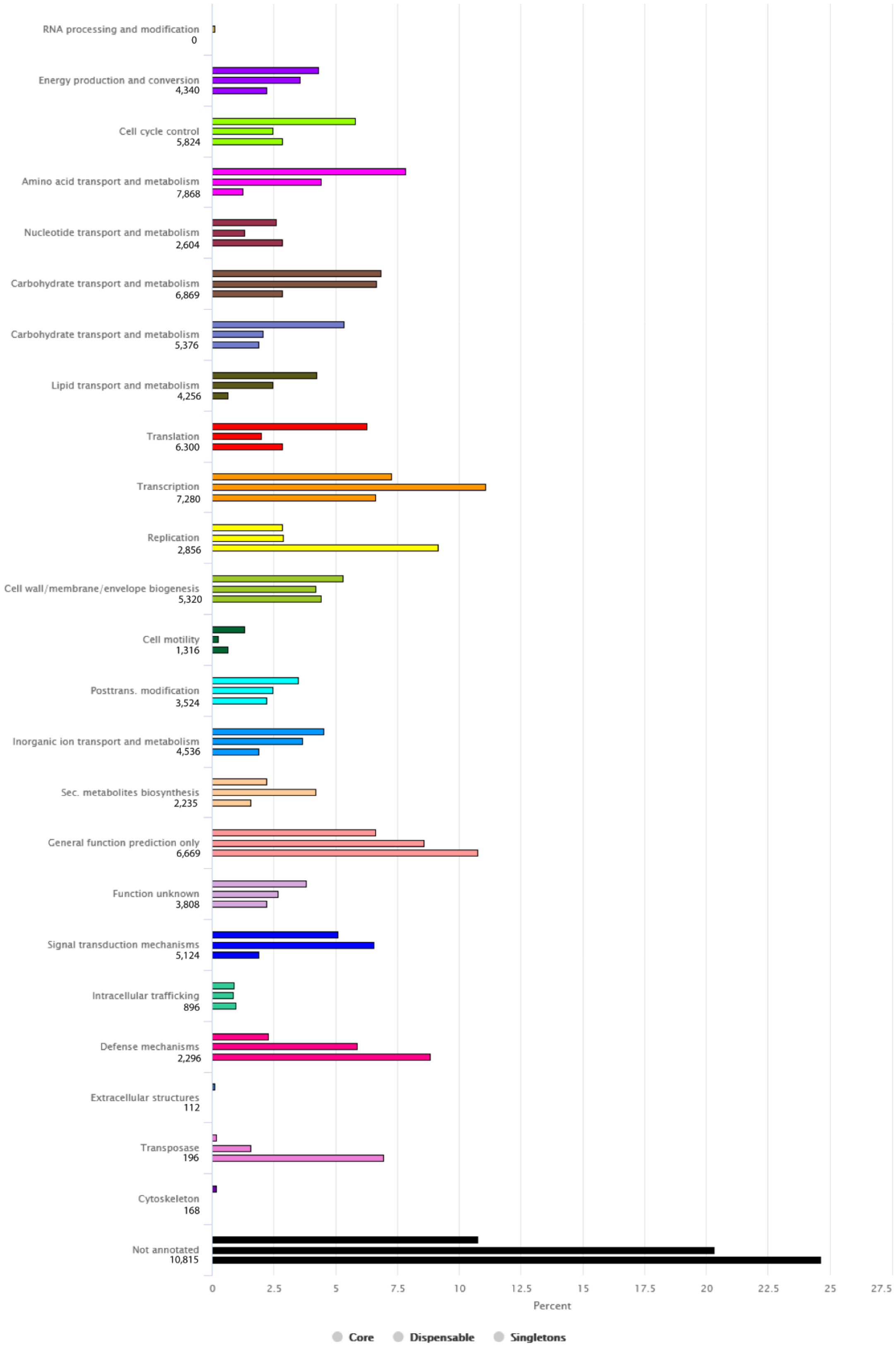

Suppl. Figure S6: Functional category analyses of the 27 *B. velezensis* isolates from Vietnamese crop plants with *B. velezensis* FZB42 as reference. 110824 COG functional categories distributed in core, dispensable, and singleton genes were found in the selected contigs.

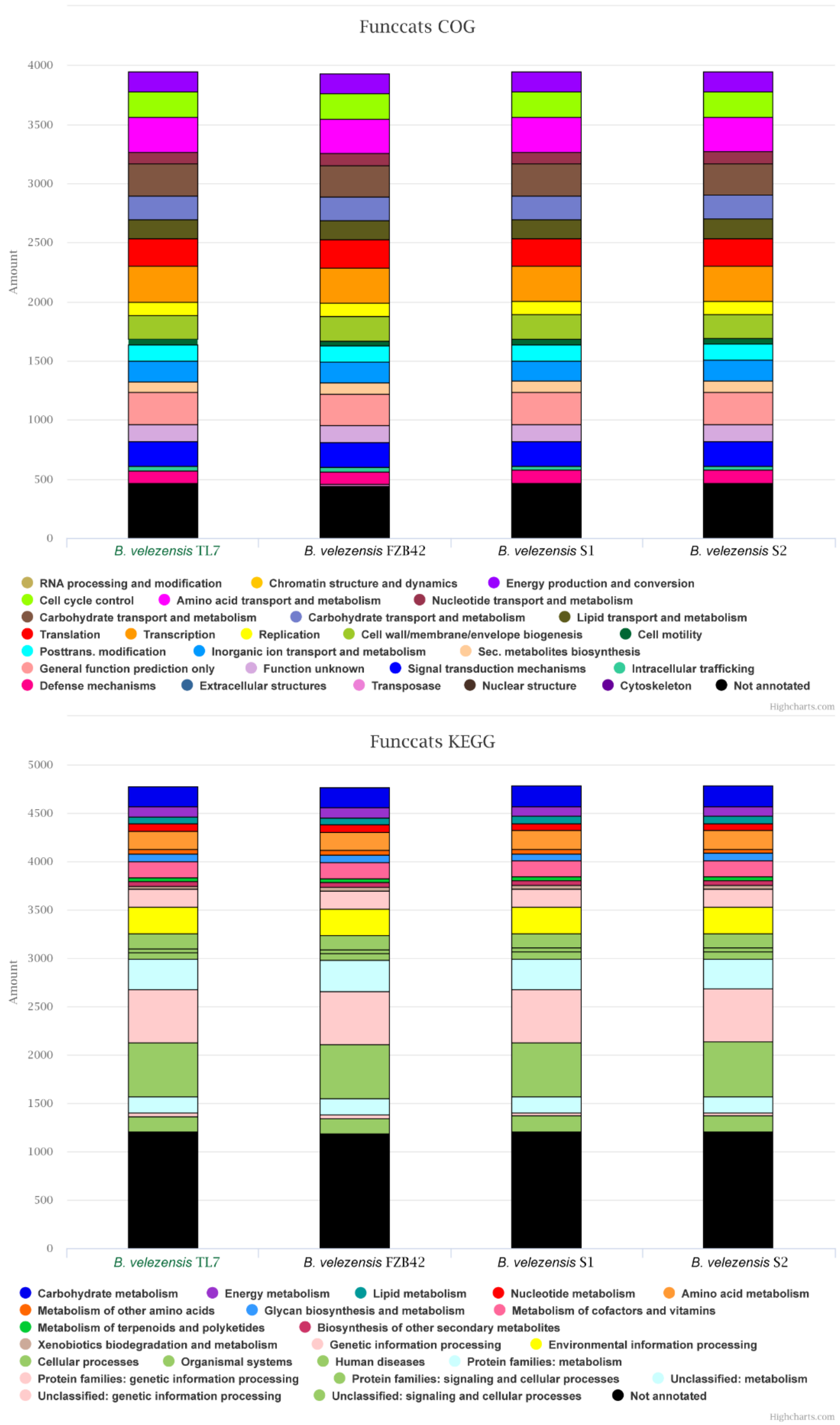

Suppl. Fig. S7: Functional category (KEGG and COG) analysis of the endophytic strain *B. velezensis* TL7 and its counterparts *B. velezensis* S1 and S2 isolated from the plant rhizosphere. For comparison, the model strain FZB42, isolated from the sugar beet rhizosphere was included. The analysis was performed with the EDGAR3.0 software package.

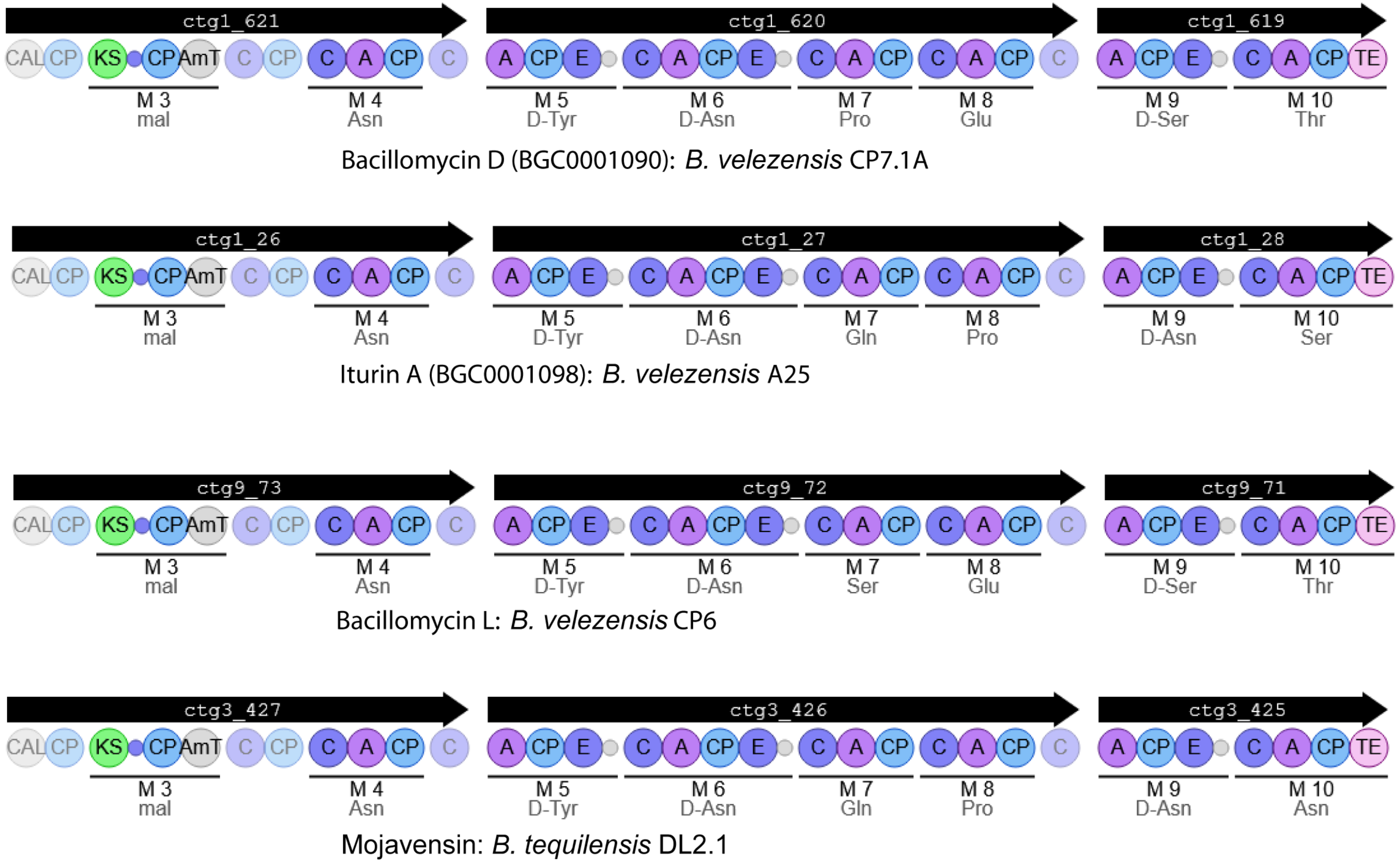

Suppl. Figure S8: Modules predicted to encode the non-ribosomal synthesis of the iturinic heptapeptides bacillomycin D, iturin A, bacillomycin L, and mojavensin were located in structurally similar gene clusters.

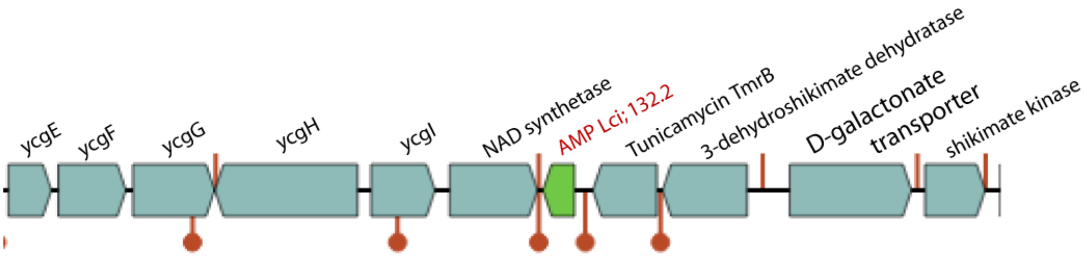

The monocistronic *lci* gene encoding the antimicrobial LCI peptide in *B. velezensis* BT2.4 (306.194-306,487)

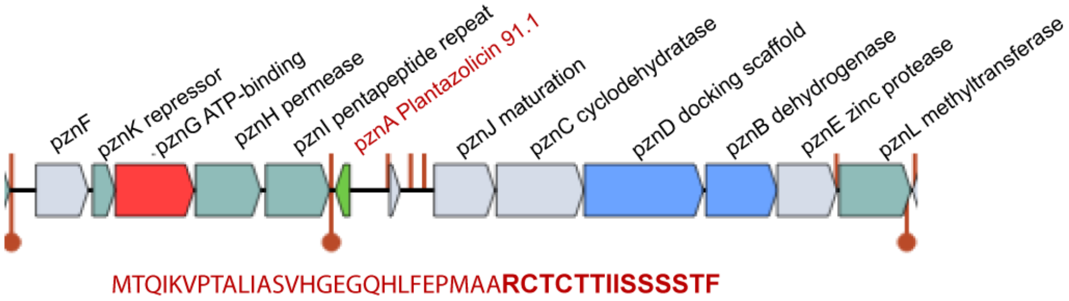

The biosynthetic plantazolicin gene cluster (BGC0000569) in *B. velezensis* BT2.4 (726,382-731,787)

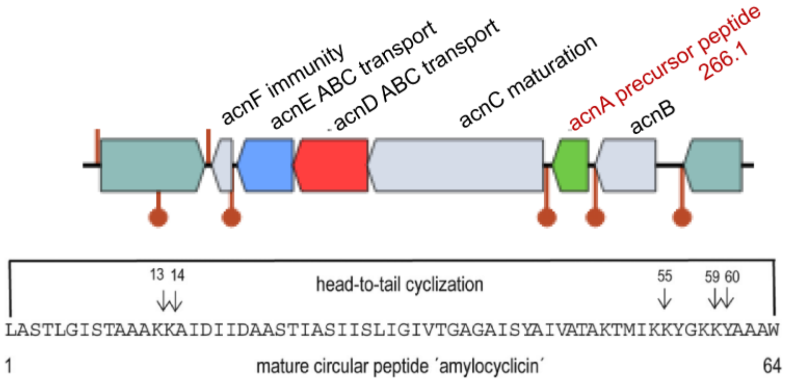

The biosynthetic amylocyclicin gene cluster (BGC0000616) in *B. velezensis* BT2.4 (3,021,491--3,036,436)

Suppl. Figure S9: Biosynthetic RiPP gene clusters (LCI, plantazolicin, amylocyclicin) occurring in the *B. velezensis* isolates closely related with FZB42

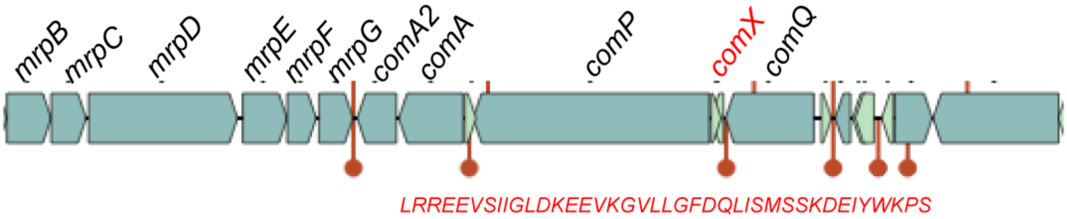

Competence pheromone ComX3; 320.1, pfam05952 (WP_012118314.1): *B. velezensis* BT2.4

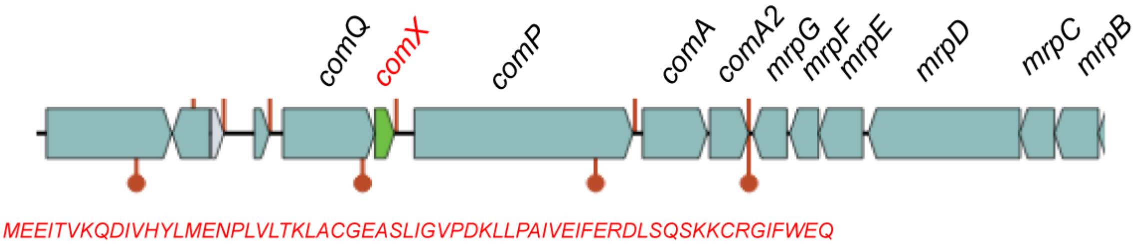

Competence pheromone ComX1; 318.1, pfam05952 (WP_206335302.1): *B. velezensis* OL1

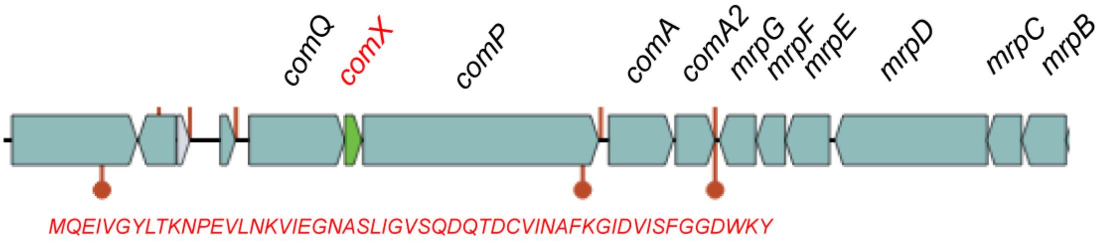

Competence pheromone ComX4; 321.1, pfam05952 (WP_007613432.1): *B. velezensis* OL1

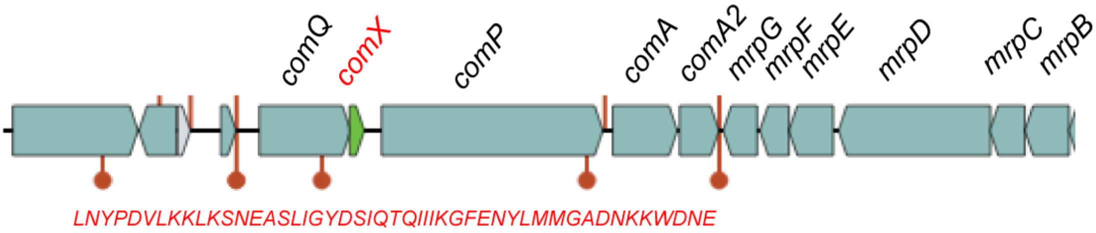

Competence pheromone ComX; 492.1, pfam05952 (WP_003152048.1): *B. velezensis* TK1

Suppl. Figure S10: Four different types of the ComX competence pheromone occurring in *B. velezensis* isolates. The *comQXP* gene clusters were detected with BAGEL4.

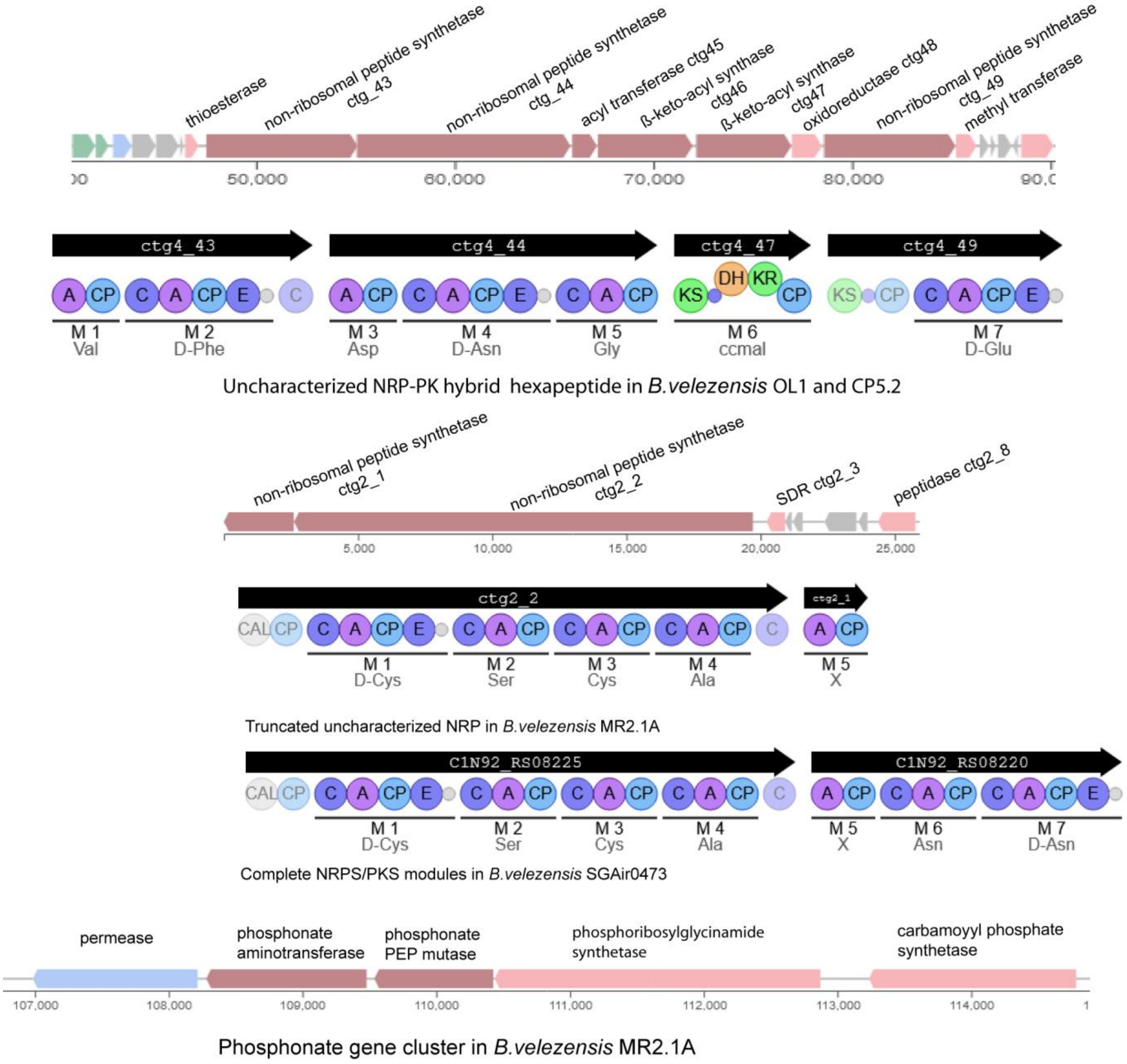

Suppl. Figure S11: Uncharacterized gene clusters in *B. velezensis* isolates predicted by antiSMASH. From top to down: (1) Uncharacterized BGC predicted to synthesize non-ribosomally the NRP-PK hexapeptide Val-D-Phe-Asp-D-Asn-Gly-ccmal-D-Glu, (2) Truncated NRP in *B. velezensis* MR2.1A. By comparison with the homologous gene cluster from *B. velezensis* SGAir0473 the sequence D-Cys-Ser-Cys-Ala-X-Asn-D-Asn can be predicted, (3) Phosphonate gene clusters were detected in *B. velezensis* MR2.1A, and *B. velezensis* EG5.1A.

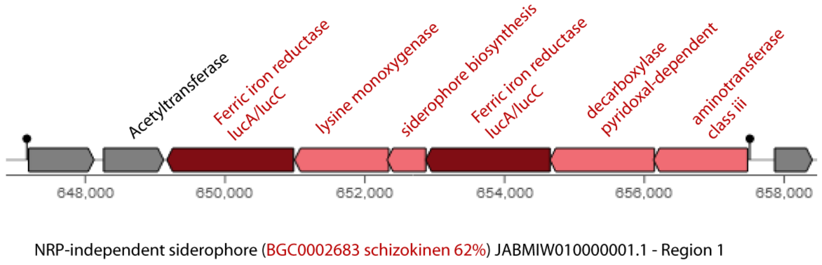

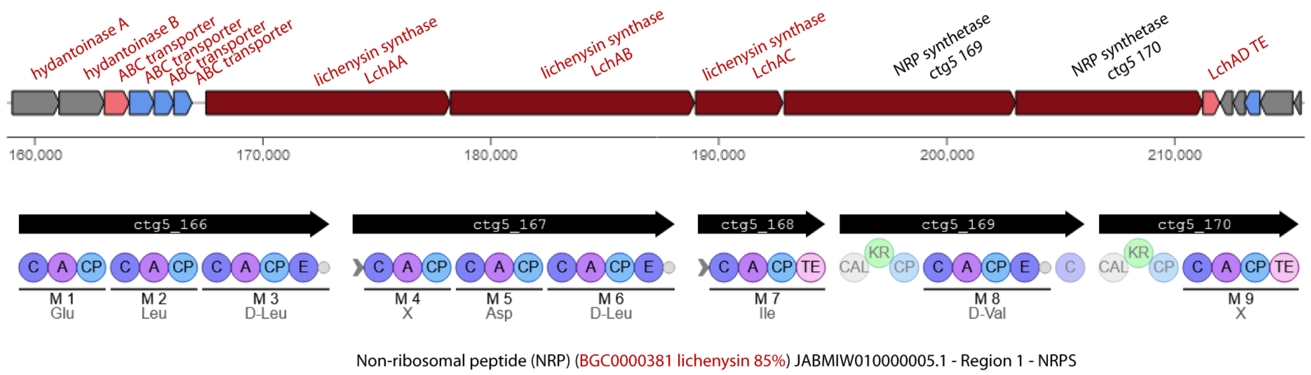

Suppl. Figure S12: Two uncharacterized biosynthetic gene clusters in *B. altitudinis* BT2.2. Top: The NRP-independent siderophore cluster contained genes with similarity to schizokinen (BGC0002683). Bottom: The nonribosomal peptide gene cluster shared genes with the surfactant lichenysin (BGC0000381) from *B.licheniformis*.

.

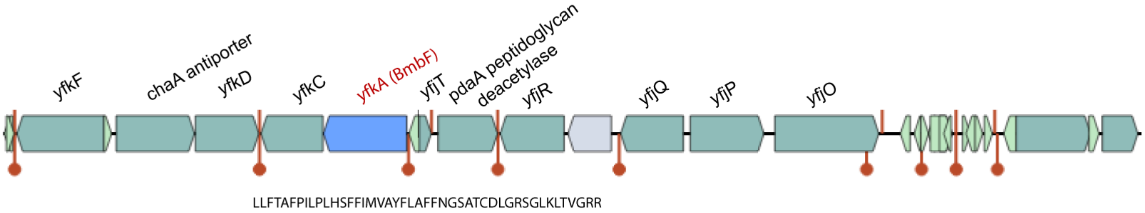

Sactipeptide (RiPP, thiopeptide), unknown, in *B. velezensis* OL1 JABSVV010000004 (187,874-207,874)

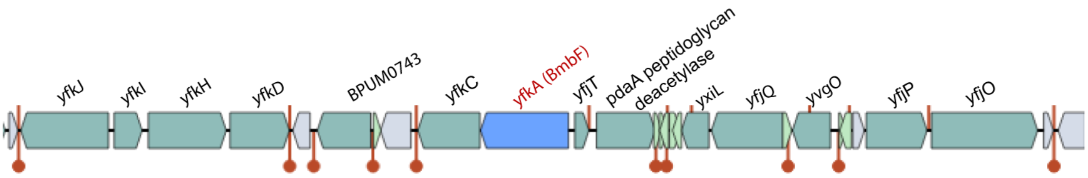

Sactipeptide (RiPP, thiopeptide), unknown, in *B. altitudinis* BT2.2 JABMIW010000006 (205,859-225,859)

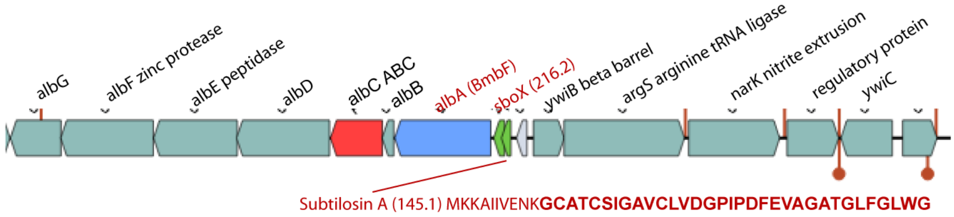

Sactipeptide (RiPP, thiopeptide) Subtilosin A (BGC0000602) in *B. tequilensis* DL2.1 JABMIY010000002 (288,098-295,038)

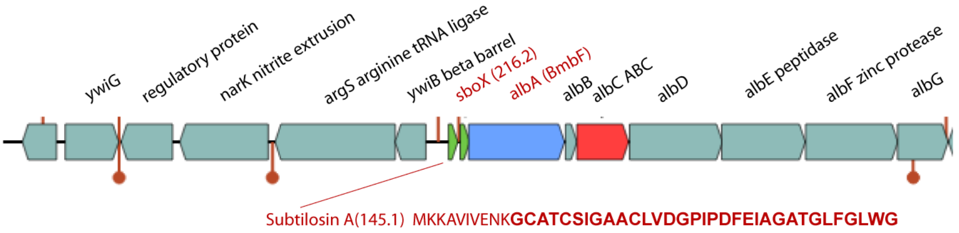

Sactipeptide (RiPP, thiopeptide) Subtilosin A (BGC0000602) in *B. subtilis* GR2.1 JABMIX010000001.1 (677,817-677,948)

Suppl. Figure S13: Sactipeptide gene clusters occurring in *B. subtilis*, *B. tequilensis*, *B. velezensis*, and *B. altitudinis* isolates. The gene clusters responsible for synthesis of the sactipeptide subtilisin A were detected in *B. subtilis* GR2.1, and *B. tequilensis* DL2.1.

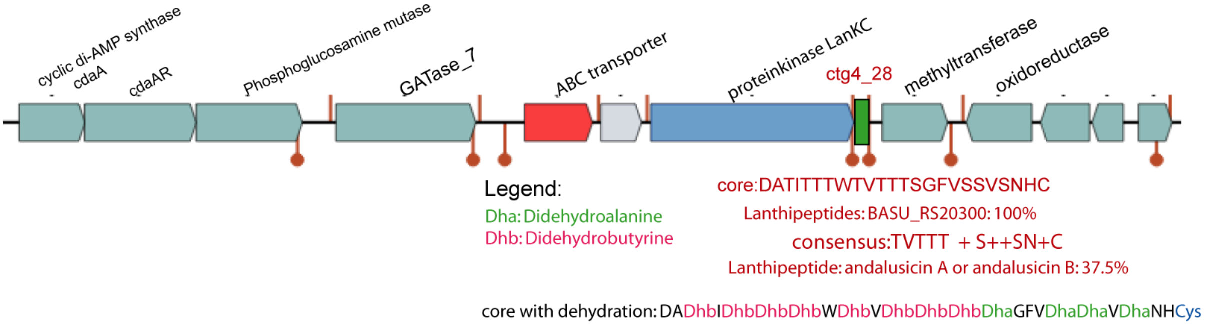

Lanthipeptide-class IV-iii:micKC (BGC0002111 andalusin A): *B. velezensis* OL1.1
JABSVV010000004: 27,333-35,205

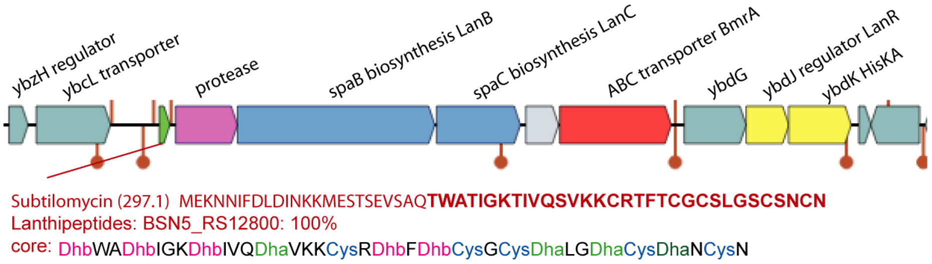

Lanthipeptide-class I LanB/LanC (BGC0000560 subtilomycin): *B. subtilis* GR2.1
JABMIX010000004: 35,021-49,367

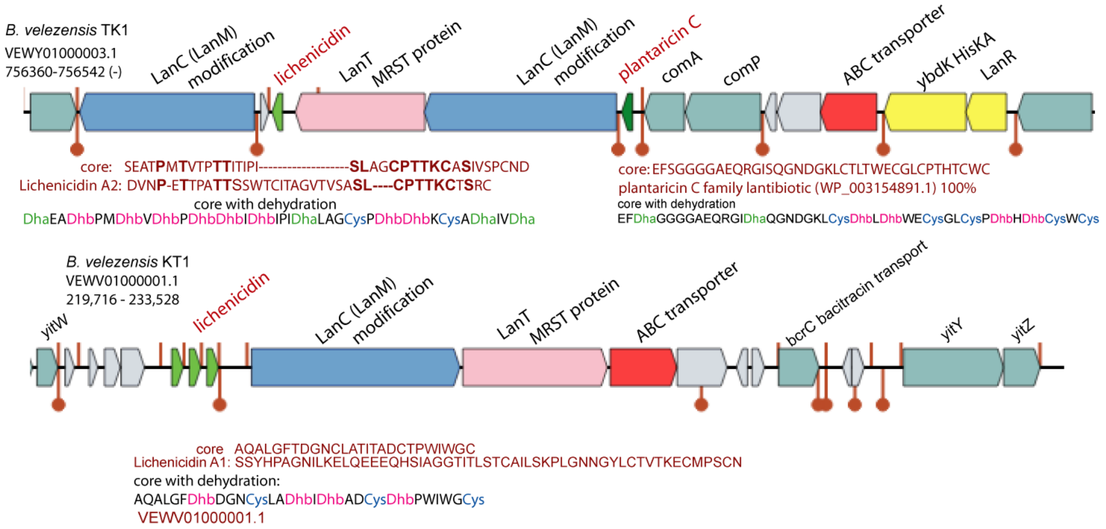

Lanthipeptide-class II (LanC-like) Lichenicidin-like: *B. velezensis* TK1 and KT1.

Suppl. Figure S14: Gene clusters responsible for synthesis of different types of lanthipeptides: Lanthipeptide class I was represented by the subtilomycin A gene cluster (BGC0000560) in *B. subtilis* GR2. *B. velezensis* harbored gene clusters for biosynthesis of the lanthipeptide classes II (TK1, KT1), and IV (OL.1.1),

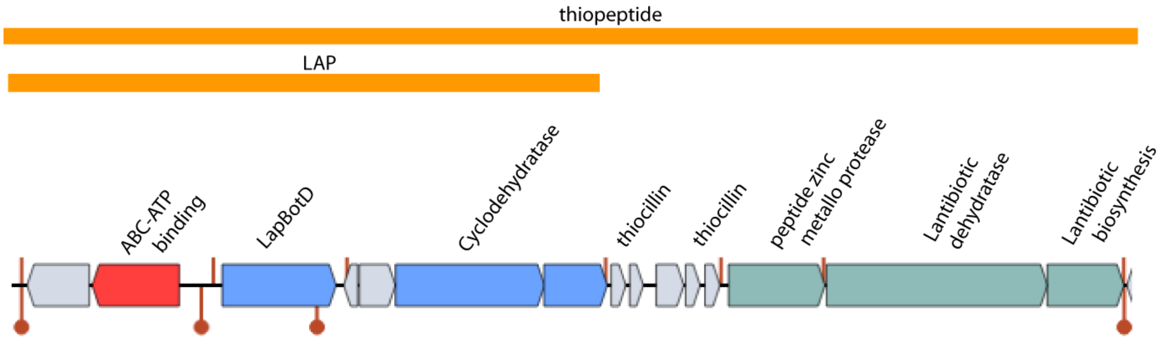

core: YSTASSFTTASCPATSASTAASASS
core with dehydrations: YDhaDhbADhaDhaFDhbDhbADhaCysPADhbDhaADhaDhbAADhaADhaDha

Suppl. Fig. S15: BGC for the thiopeptide thiocillin (*B. velezensis* CP6)

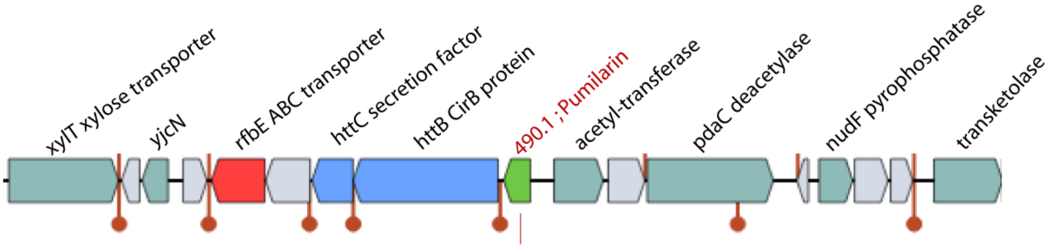

Pumilarin (490.1) MTETRNEIKLHVLFGALAVGFLMLALFSFSLQVLPVADLAKEFGIPGSVAAVVLNVVEAGGAVTTIVSILTAVGSGGLSLIAAAGKETIRQYLKNEIKKKGRKAVIAW
Subclass Head-to-tailcyclizedpeptides

The circular head-to-tail cyclized pumilarin in *B. altitudinis* BT2.2 JABMIW010000002 (417,257-417,583)

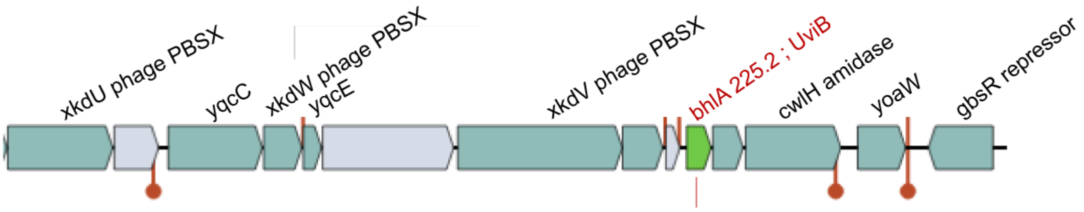

MVKENKFSKIFILMALSFLGLALFSASLQFLPIAHMAKEFGIPAAVAGTVLNVVEAGGWVTTIVSILTAVGSGGLSLLAAAGRESIKAYLKKEIKKKGKRAVIAW
MULTISPECIES: BhlA/UviB family holin-like peptide [Bacillus] WP_008344062.1) 98%
Head to tail cyclized BGC0000489 enterocin AS-48 biosynthetic gene cluster from *Enterococcus faecalis* 71%

The circular head-to-tail cyclized BhlA/UviB peptide (225.2) resembling enterocin AS-48 (BGC0000489) *B. altitudinis* BT2.2 JABMIW010000004 (377,156-392,734).

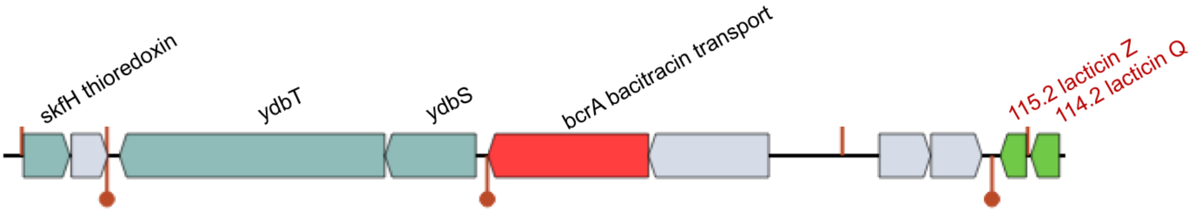

WP_017358896.1 aureocin A53 family class IId MATFLQLVAQLTAKQAI**WAWANK**KKVLEWIGQGFSFKWISNQIIRALAG: 100%
WP_058206662.1 leaderless class II bacteriocin lacticin Q/lacticin Z (Lactococcus lactis): 41.18%

The class II bacteriocin aureocin A53 (114.2/115.2) resembling lacticin Q/lacticin Z *B. altitudinis* BT2.2 JABMIW010000010.1 (27833-38198)

Suppl. Figure S16: Known and unknown RiPPs in *B. altitudinis* BT2.2. The head-to-tail cyclized pumilarin resembles amylocyclicin in *B. velezensis*. Another head-to-tail cyclized peptide (BhlA/UviB family) was similar to enterocin-48 from *Enterococcus lactis*. The leaderless class II bacteriocin aureocin A53 was similar to lacticin from *Lactococcus lactis*.
